# Immunogenomic Gene Signature of Cell-Death Associated Genes with Prognostic Implications in Lung Cancer

**DOI:** 10.3390/cancers13010155

**Published:** 2021-01-05

**Authors:** Pankaj Ahluwalia, Meenakshi Ahluwalia, Ashis K. Mondal, Nikhil Sahajpal, Vamsi Kota, Mumtaz V. Rojiani, Amyn M. Rojiani, Ravindra Kolhe

**Affiliations:** 1 Department of Pathology, Medical College of Georgia, Augusta University, Augusta, GA 30912, USA; pahluwalia@augusta.edu (P.A.); mahluwalia@augusta.edu (M.A.); amondal@augusta.edu (A.K.M.); nsahajpal@augusta.edu (N.S.); mrojiani@augusta.edu (M.V.R.); AROJIANI@augusta.edu (A.M.R.); 2 Department of Medicine, Medical College of Georgia, Augusta University, Augusta, GA 30912, USA; vkota@augusta.edu

**Keywords:** lung cancer, LUAD, gene expression, prognostic genes, cell death, apoptosis, necrosis, tumor microenvironment, immunotherapy

## Abstract

**Simple Summary:**

The human body consists of trillions of cells and several million of them die daily. These natural processes which determine the fate of a cell in the human body can be broadly defined as programmed cell death (apoptosis and autophagy) and a non-programmed, passive cell death (necrosis). The inherent genetic diversity in humans and differential expression of mRNAs belonging to these cell death pathways can provide clinically actionable information. In this study, we have discovered a differential 21-gene cell death signature that significantly separates lung cancer patients based on their survival. The patients with increased expression of this genomic signature were found to be at higher risk of dying early. Interestingly, this patient group showed significant perturbations in the expression of cytokines and infiltration of immune cells within these tumors. Therefore, the discovery of this novel genomic signature can be used for prognostication of lung cancer patients, and most importantly we can tailor personalized novel immunotherapies for their treatment.

**Abstract:**

Lung cancer is one of the leading causes of death worldwide. Cell death pathways such as autophagy, apoptosis, and necrosis can provide useful clinical and immunological insights that can assist in the design of personalized therapeutics. In this study, variations in the expression of genes involved in cell death pathways and resulting infiltration of immune cells were explored in lung adenocarcinoma (The Cancer Genome Atlas: TCGA, lung adenocarcinoma (LUAD), 510 patients). Firstly, genes involved in autophagy (*n* = 34 genes), apoptosis (*n* = 66 genes), and necrosis (*n* = 32 genes) were analyzed to assess the prognostic significance in lung cancer. The significant genes were used to develop the cell death index (CDI) of 21 genes which clustered patients based on high risk (high CDI) and low risk (low CDI). The survival analysis using the Kaplan–Meier curve differentiated patients based on overall survival (40.4 months vs. 76.2 months), progression-free survival (26.2 months vs. 48.6 months), and disease-free survival (62.2 months vs. 158.2 months) (Log-rank test, *p* < 0.01). Cox proportional hazard model significantly associated patients in high CDI group with a higher risk of mortality (Hazard Ratio: H.R 1.75, 95% CI: 1.28–2.45, *p* < 0.001). Differential gene expression analysis using principal component analysis (PCA) identified genes with the highest fold change forming distinct clusters. To analyze the immune parameters in two risk groups, cytokines expression (*n* = 265 genes) analysis revealed the highest association of *IL-15RA* and *IL 15* (> 1.5-fold, *p* < 0.01) with the high-risk group. The microenvironment cell-population (MCP)-counter algorithm identified the higher infiltration of CD8+ T cells, macrophages, and lower infiltration of neutrophils with the high-risk group. Interestingly, this group also showed a higher expression of immune checkpoint molecules *CD-274 (PD-L1)*, *CTLA-4*, and T cell exhaustion genes *(HAVCR2*, *TIGIT*, *LAG3*, *PDCD1*, *CXCL13*, and *LYN*) (*p* < 0.01). Furthermore, functional enrichment analysis identified significant perturbations in immune pathways in the higher risk group. This study highlights the presence of an immunocompromised microenvironment indicated by the higher infiltration of cytotoxic T cells along with the presence of checkpoint molecules and T cell exhaustion genes. These patients at higher risk might be more suitable to benefit from PD-L1 blockade or other checkpoint blockade immunotherapies.

## 1. Introduction

Lung cancer is one of the leading causes of cancer-related mortality worldwide [1]. Lung cancer is divided mainly into two subtypes: small lung cancer (SCLC) and non-small cell lung cancer (NSCLC). NSCLC accounts for the majority (around 85%) of all lung cancer cases and includes two major types. Among NSCLC, lung adenocarcinoma (LUAD) and lung squamous cell carcinoma (LUSC) form 70% and 30% of all the total cases, respectively [2]. Despite recent advances in surgery, chemotherapy, radiotherapy, and immunotherapy, the 5-year survival of lung cancer patients remains dismally poor [3]. Therefore, novel prognostic methods to identify patients at higher risk are required that can further assist in the design of new therapeutic options for LUAD patients.

The tumor microenvironment (TME) consists of tumor cells and the surrounding area which includes blood vessels, cytokines, chemokines, fibroblasts, extracellular matrix [4]. Cell death is an essential process that is necessary for the growth and development of an organism. There are diverse cell death processes that are initiated in the tumor microenvironment due to the normal biological response, external stimuli, or response to therapies. The resulting immune response due to alterations in the death activity of the tumor, stromal, endothelial, and immune cells can significantly alter the trajectory of tumor growth [5]. There are three classical cell death pathways: autophagy, apoptosis, and necrosis [6]. Autophagy is the degradation of cellular macromolecules to generate metabolites in the situation of cellular stress. Autophagy results in the formation of autophagosome, a lipid bilayer vesicle which fuses with lysosomes for the degradation of cellular organelles, proteins, etc. [7]. Autophagy is generally a pro-survival mechanism but the chronic induction of stress can cause irreversible damage which might lead to apoptosis or necrosis [8,9]. Apoptosis is a programmed cell death that mediates through two pathways: the mitochondria-mediated intrinsic pathway and extrinsic pathway involving death receptors (DRs) [10]. Its characteristics features are nuclear fragmentation, membrane blebbing, and chromosomal condensation [11]. Necrosis is a form of cell death that is non-physiological, non-specific and induced by stress [12]. Apoptosis is the active dismantling of cells to prevent the release of inflammatory mediators whereas necrosis results in the disruption of the cellular membrane, spilling inflammatory cellular contents into the tumor microenvironment [13,14]. The dysregulation of cell death programs can play a significant role in tumorigenesis [15].

NSCLC is one of the most heterogenous tumors with a varying degree of aggressiveness between its subtypes which require different treatment regimens [16]. In the absence of activating ALK and EGFR mutations or ROS1 translocation, the first line of treatment generally includes platinum-based chemotherapy [17]. The response rate of these therapies is only between 15 and 30% [18]. Furthermore, 30% of the patients who experience disease progression are provided with second-line therapies which include pemetrexed and docetaxel along with EGFR TKI, erlotinib and gefitinib [19]. In 2015 and 2016, immune checkpoint inhibitors targeting PD-1/PD-L1 axis, pembrolizumab and nivolumab were approved by the FDA as therapeutic approaches [17]. Although immunotherapies have begun to evolve as an attractive approach, the prognostic and predictive identification of patients responsive to these immunotherapies are generally lacking [18]. Furthermore, the benefits of these immunotherapies are not observed in all the patients because of the variability in the patients [20]. Thus, new biomarkers or risk stratification methods are required which could assist in the clinical management of LUAD patients.

The advancement of multi-omic analysis and differential expression profiles have identified new prognostic biomarkers for LUAD patients [21,22]. Most of these bioinformatics studies are mathematical analyses of whole-scale genetic or transcriptomic data which lack specialized focus on biological pathways [22]. In this study, LUAD patients were stratified based on cell death-based gene signatures along with the characterization of their immune response.

## 2. Methods

### 2.1. Gene Expression Analysis to Determine Cell Death Index (CDI)

To identify the prognostic association of cell death genes in lung adenocarcinoma, the Kyoto Encyclopedia of Genes and Genomes (KEGG) and Gene Ontology (GO) databases were accessed through the Gene Set Enrichment Analysis (GSEA) website to access the relevant gene lists (https://www.gsea-msigdb.org). The KEGG dataset of autophagy (*n* = 34 genes), apoptosis (*n* = 86 genes) and GO gene list of necrosis (*n* = 49 genes) were downloaded. These genes were analyzed in cBioportal to quantify the perturbations in The Cancer Genome Atlas: TCGA—lung adenocarcinoma (LUAD) (https://www.cbioportal.org/) (TCGA-LUAD RNA Seq, V2 *n* = 510 patients). cBioportal is an online platform to analyze aberrations and variations in gene expression across all major cancers analyzed in the TCGA project [23]. Survival information of individual genes was analyzed using a log rank test on *n* = 510 patients. Subsequently, 150 patients in each cell death group were picked based on the higher or lower expression of specific cell death pathway genes (75 with the highest z-score and 75 with the lowest z-score in all the individual pathways). Additionally, significant genes from each cell death pathway were selected to generate a combined 21 gene signature to form a cell death index (CDI). From 510 patients, 59 patients showing the highest expression of cell death genes (CDI high group) and 49 patients with the lowest expression of cell death genes (CDI low group) were selected for further analysis. Kaplan–Meier survival analyses were performed to compare the overall survival (OS), disease-free survival (DFS), disease-specific survival (DSS) and progression-free survival (PFS) data in the two cohorts.

### 2.2. Clinico-Pathological Analysis

Clinico-pathological characteristics of lung cancer patients were downloaded from TCGA (https://gdc.cancer.gov/). The numeric values were split at the median and compared between high- and low-risk groups. Pearson’s chi-square (χ^2^) test was used to compare these sets of categorical variables.

### 2.3. Cox-Proportional Hazard

Univariable and multivariable Cox proportional hazards models were used to analyze clinico-pathological variables (CDI, tumor stage, lymph node, distant metastasis, age, sex, and radiation therapy). The hazard ratios (HR) with 95% confidence intervals (CI) were based on overall survival (OS).

### 2.4. Differential Expression of Genes and Principal Component Analysis (PCA)

RNA-Seq data of TCGA-LUAD RNA Seq, V2 *n* = 510 patients, were downloaded from the National Cancer Institute portal (https://gdc.cancer.gov/). DESeq2, a R programming statistical package was used to analyze differentially expressed genes between the high CDI and low CDI group (https://bioconductor.org/packages/DESeq2.html) [24].

### 2.5. Immune Cell Infiltration Analysis

There are multiple computational deconvolution methods that can be utilized to quantify the proportion of immune cells from heterogenous samples [25]. In this study, the microenvironment cell-population (MCP)-counter method was used to quantify the infiltration of immune cells using the TIMER: Tumor IMmune Estimation Resource portal (http://timer.cistrome.org/) [26]. The MCP-counter algorithm estimates the number of infiltrated immune and stromal cells in the samples by quantifying cell-specific transcripts. These signatures are validated using RNA mixtures and immunohistochemistry (IHC) measurements [27]. In this study, normalized RNA-Seq values of patients in High CDI and Low CDI patients were used as input for the MCP-counter algorithm using the TIMER portal. MCP-counter has been utilized in several gene expression studies to quantify the abundance of immune cells in diverse samples [28,29,30,31,32].

### 2.6. Evaluation of Cytokines, Checkpoint Molecules and T Cell Exhaustion Genes

To evaluate immune activity in patients, the z-scores of different cytokines were downloaded from cBioportal. Cytokine genes information were downloaded using keyword: ‘KEGG cytokine-cytokine receptor interaction’ (*n* = 265 genes) (https://www.gsea-msigdb.org) (Table S6) [33]. Functional enrichment analysis was performed using string-DB portal (https://string-db.org/). The T cell exhaustion markers genes were analyzed as previously published (*HAVCR2*, *TIGIT*, *LAG3*, *PDCD1*, *CXCL13*, *LAYN*) [34].

### 2.7. Functional Enrichment Analysis

Gene level functional enrichment was performed using g:Profiler program (https://biit.cs.ut.ee/gprofiler) [35]. It quantifies and maps genes to the corresponding enriched pathways based on statistical significance. The data sources used for this analysis included: Gene Ontology (GO): Molecular Function (MF), GO: Biological Processes (BP), GO: Cellular Components (CC), Kyoto Encyclopedia of Genes and Genomes (KEGG), Reactome (REAC), WikiPathways (WP), Transfac (TF), miRTarBase (MIRNA), Human Protein Atlas (HPA), CORUM protein complexes (CORUM) and Human Phenotype Ontology (HP).

### 2.8. Statistical Analysis

The statistical significance of OS, DFS, PFS and DSS was computed using the Long-rank t-test through cBioportal. For the comparison of clinico-pathological parameters, Pearson’s chi-square (χ^2^) test was applied (*p* < 0.05). The DEGs were analyzed by the DEseq2 package in R. The results were interpreted in R using ‘plotPCA’ function for Principal component analysis and volcano plot was generated using ‘enhancedvolcano’ package (http://bioconductor.org/packages/EnhancedVolcano.html). The cytokines z-scores were compared between each group using an unpaired t-test corrected for multiple comparisons using Holm–Sidak method (*p* < 0.05). For the comparison of immune cells and T cell exhaustion markers, unpaired t-test with Welch correction was used (*p* < 0.05). The statistical analyses were performed using R (R Foundation for Statistical Computing, Vienna, Austria, version 3.6.1) (http://www.R-project.org/), JMP-Pro (version 14.0.0, SAS Institute, Cary, NC, USA) and GraphPad Prism (version 8 GraphPad Software, La Jolla, CA, USA). P values < 0.05 were considered statistically significant.

## 3. Results

### 3.1. Survival Analysis of Patients Using Cell Death Index (CDI)

**Figure 1:**
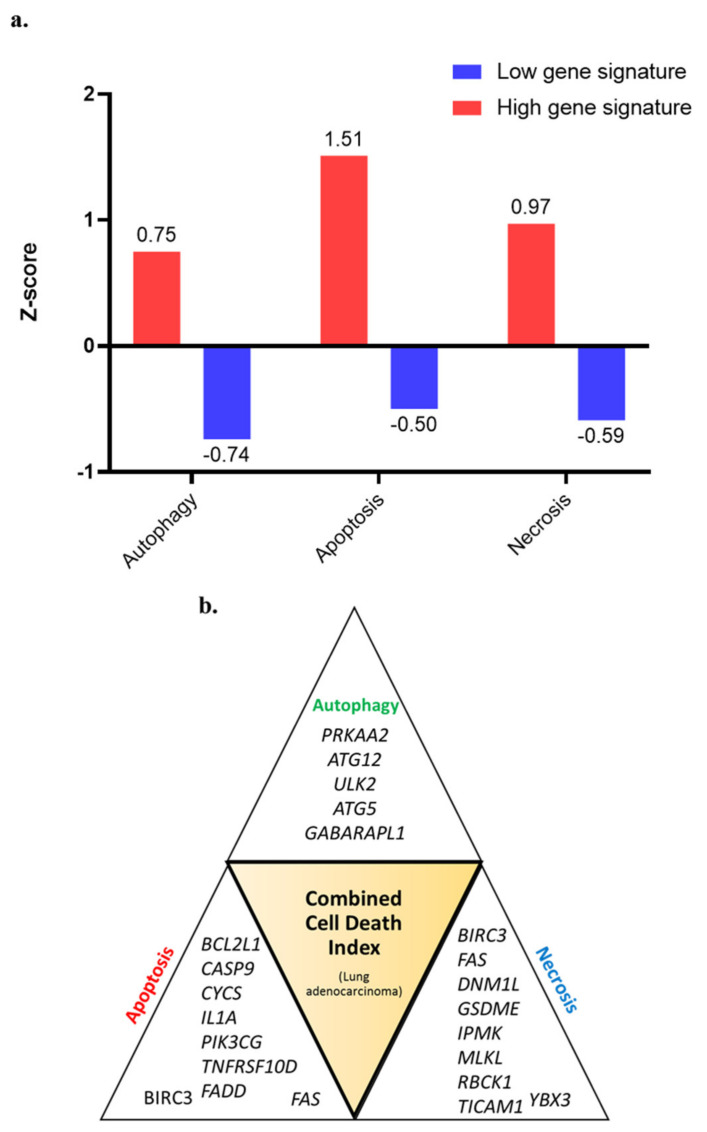
(**a**) Difference in the median z-scores in each cell death pathway (autophagy, apoptosis and necrosis) identifying patients with the higher expression (z-score); (**b**) combined cell death index (CDI) was generated, which included patients with the highest expression of genes involved in autophagy, apoptosis, and necrosis.

Higher expression of genes was studied for prognostic association with OS, DFS, DSS and PFS. In Autophagy gene list (*n* = 34 genes) *ATG12*, *GABARAPL1*, *IFNA17*, *IFNA8* showed association with survival (Table S1) (*p* < 0.05). For apoptosis (*n* = 86 genes), *BCL2L1*, *CASP9*, *CHP2*, *CYCS*, *EXOG*, *IL1A*, *IL1R1*, *IL1RAP*, *IL3RA*, *NFKBIA*, *PIK3CA*, *PIK3CD*, *PIK3CG*, *PIK3R1*, *PIK3R2*, *PRKAR1B*, *TNFRSF10A*, *TNFRSF10B*, *TNFRSF10D*, *TNFRSF1A* showed association with survival (*p* < 0.05) survival (Table S2). For necrosis, (*n* = 49 genes), *DNML1*, *GSDME*, *IPMK*, *MLKL*, *RBCK1*, *TICAM1*, *YBX3* showed associated with survival (Table S3). *BAX*, *BIRC3*, *FADD* and *FAS* overlapped between apoptosis and necrosis. To identify the multi-gene prognostic signature of individual cell death pathway, various combinations of genes were tried which resulted in three separate gene signatures. In combination, the five-gene autophagy signature, nine-gene apoptosis signature, and seven-gene necrosis signature showed prognostic association with lung cancer (Table 1). The z-scores of patients showing the highest and lowest expression of these gene signatures were split into two groups and the differences in the median z scores of autophagy (0.75 and −0.74), apoptosis (1.51 and −0.50), necrosis (0.97 and −0.59) (Figure 1a). The patients which were included in ≥2 cell-death pathways were selected and split based on gene expression CDI (Figure 1b).

### 3.2. RNA-Seq Analysis of Patients

**Figure 2:**
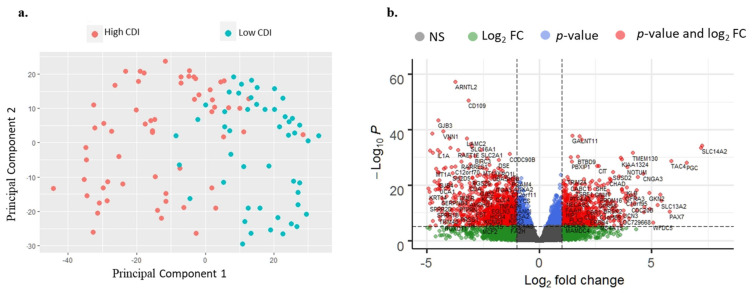
(**a**) Principal component analysis (PCA) showing distinct clustering between the RNA-seq expression of high CDI patients compared to low CDI patients; (**b**) volcano plot showing the differential expression of genes between high CDI and low CDI (*p* < 0.01, log_2_ fold-change > 2).

There were 943 differentially expressed genes, of which 329 genes were upregulated at >2-fold in high-risk patients compared to the lower risk group (Table S4). In the low-risk group, a total of 614 genes were upregulated at >2-fold compared to the high-risk group (Table S5). The principal component analysis (PCA) showed a distinct separation between the high-risk group and low-risk group based on CDI (Figure 2a). The volcano plot of differentially expressed genes between high-risk and low-risk patients is depicted in Figure 2b.

### 3.3. Clinico-Pathological and Survival Analysis

Among clinical parameters, stage, lymph node involvement, aneuploidy score, survival status (OS, PFS, DFS, DSS) were found to be significantly different in the high and low-risk group (Table 2a). In univariate Cox proportion hazard analysis, the high-risk group was associated with worse survival (Hazard ratio: H.R 1.75, 95% CI: 1.28–2.45, *p* < 0.001). Other significant variables associated with poorer survival were stage III + IV group patients (H.R 2.68, 95% CI: 1.95–3.63, *p* < 0.001), patients with the tumor spread to lymph node (N1 + N2 + N3) (H.R 2.61, 95% CI: 1.94–3.52, *p* < 0.001), patients with distant metastasis (H.R 2.12, 95% CI: 1.19–3.52, *p* < 0.01) and patients with radiation therapy (H.R 2.02, 95% CI: 1.36–2.90, *p* < 0.001). In multivariate Cox proportion hazard, only three variables showed association with poorer survival: -High CDI (H.R 1.62, 95% CI: 1.11–2.36, *p* < 0.01), stage III + IV group patients (H.R 2.13, 95% CI: 1.39–3.25, *p* < 0.001), and patients with lymph node spread (N1 + N2 + N3) (H.R 2.25, 95% CI: 1.59–3.19, *p* < 0.001).

**Figure 3:**
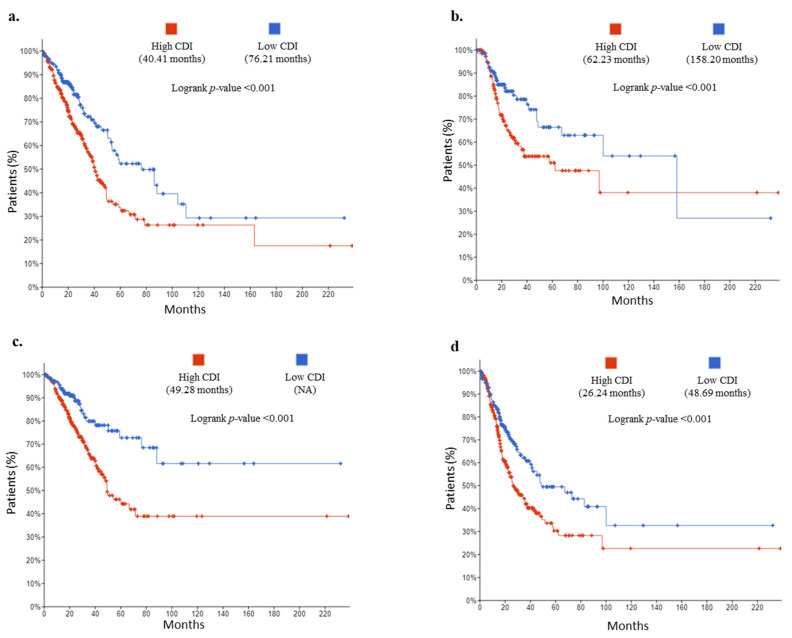
Kaplan–Meier curves of patients with high CDI vs. low CDI: (**a**) overall survival (OS), (**b**) disease-free survival (DFS), (**c**) disease-specific survival (DSS), (**d**) progression-free survival (PFS).

Using Kaplan–Meier analyses, patients in the high-risk group were differentiated from low-risk based on CDI. The overall survival difference observed in both the risk groups for OS was 40.41 vs. 76.21 months (log-rank test, *p* < 0.001) (Figure 3a). For DFS, 62.23 vs. 158.20 months (log-rank test, *p* < 0.001) (Figure 3b), for DSS 49.28 high-risk patients (low CDI patients did not reach the survival months threshold) (log-rank test, *p* < 0.001) (Figure 3c), and for PFS, 26.24 vs. 48.69 months (log-rank test, *p* < 0.001) (Figure 3d).

### 3.4. Cytokine Gene Expression Analysis

**Figure 4:**
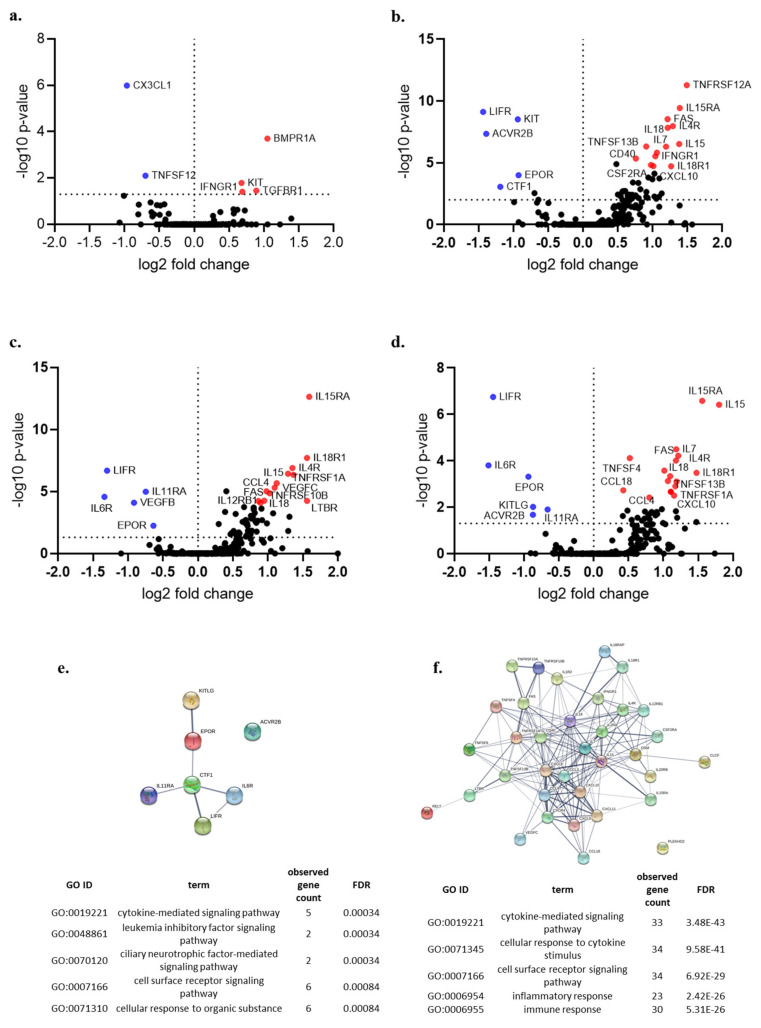
Volcano plot showing the differential expression of cytokines (*n* = 265 genes) between patients in the high and low gene expression of individual cell death pathways (*p* < 0.05) (**a**) autophagy, (**b**) apoptosis, (**c**) necrosis, (**d**) cell death index (CDI); (**e,f**) Gene Ontology (GO) functional enrichment of genes with higher expression in low CDI and high CDI group, respectively, (False Discovery Rate: FDR).

In the autophagy group, the expression of *BMPR1A*, *KIT*, *TGFBR1* and *IFNGRI* was found to be higher in the high-risk group, whereas the expression of *CX3CL1* and *TNFSF11* was found to be higher in a low-risk group (Figure 4a). Similar analyses were performed on the apoptosis and necrosis group (Figure 4b,c). In the CDI group, the expression of *IL15RA*, *IL-15*, *IL-7*, *IL4R*, *IL-18*, *FAS*, *TNFSF13B*, *TNFSRF1A*, *CXCL10* among others were found to be higher in patients in a high-risk group whereas, the expression of *LIFR*, *IL6R*, *EPOR*, *KITLG*, *ACVR2B* and *IL11RA* was found to be higher in the low-risk group (Figure 4d). Functional enrichment analysis revealed a higher proportion of inflammatory cytokines in the high CDI group (Figure 4e,f).

### 3.5. Immune Cell Analysis

**Figure 5:**
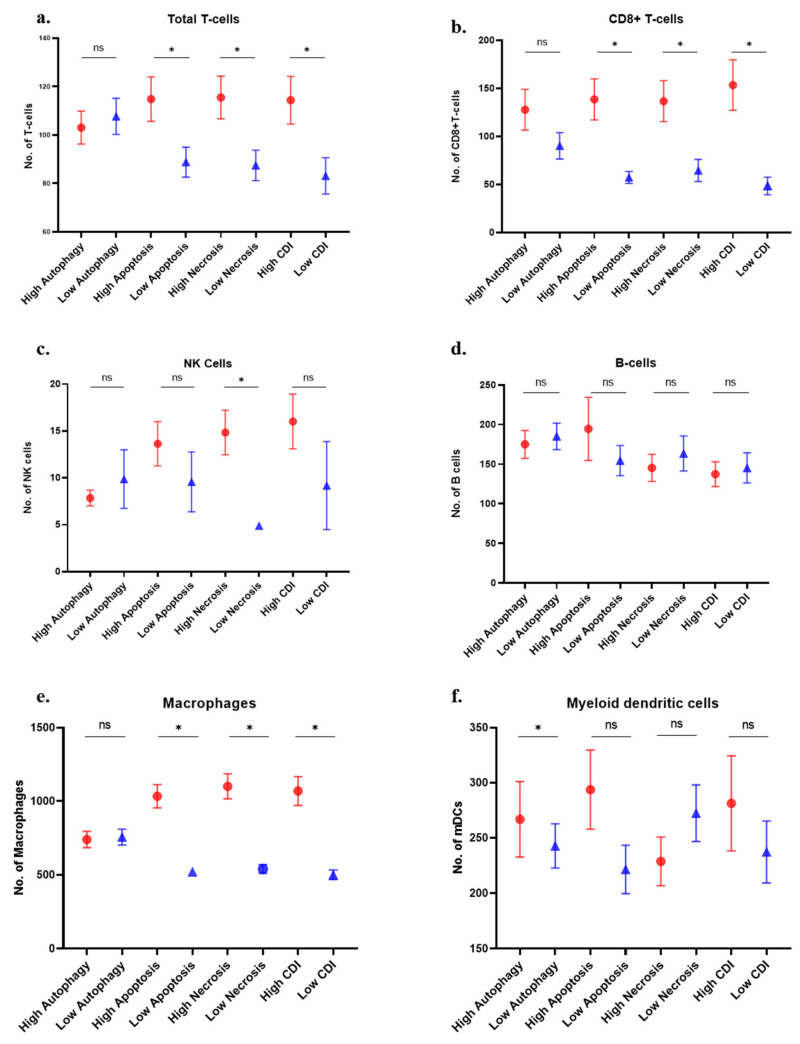

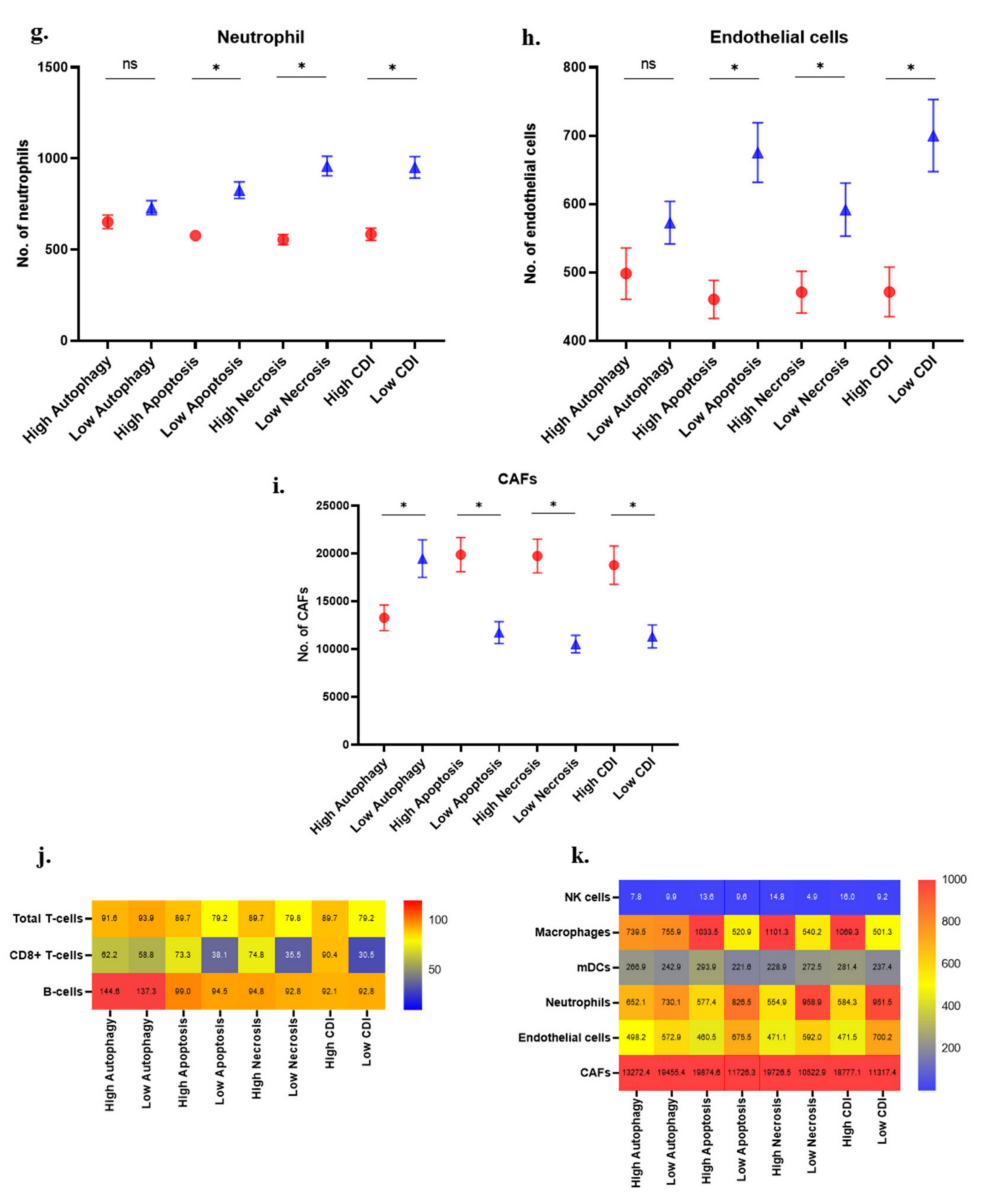
Immune cell distribution in patients with the high and low gene expression of individual cell death pathways: (**a**) total T cells; (**b**) CD8+ T cells; (**c**) natural killer cells; (**d**) B cells; (**e**) macrophages; (**f**) myeloid dendritic cells; (**g**) neutrophils; (**h**) endothelial cells; (**i**) cancer-associated fibroblast (CAFs); (**j**) heatmap of lymphocytes distribution in cell death groups (median values); and (**k**) the heatmap of other immune cells in different cell death groups (median values) (* *p* < 0.05).

MCP-counter algorithm demonstrated that greater T cell and CD8+ T cell infiltration correlated with high apoptosis, necrosis, and high cell death index patient groups (Figure 5a,b). NK cells infiltration was found to be higher in patients with high necrosis (Figure 5c). B cells infiltration did not show any significance with any group (Figure 5d). Macrophages showed higher infiltration with high apoptosis, necrosis, and high CDI group (Figure 5e). Myeloid dendritic cells showed significantly higher infiltration in high autophagy group only (Figure 5f).

Neutrophils showed higher infiltration in all the groups except the autophagy group (Figure 5g). Endothelial cells and cancer-associated fibroblasts showed higher presence in the high cell death groups except for endothelial cells in the autophagy group (Figure 5h,i). These differences are presented in the heatmap of lymphocytes and other cells in different cell death groups (Figure 5j,k).

### 3.6. Immune Suppression and T-Cell Exhaustion Markers

**Figure 6:**
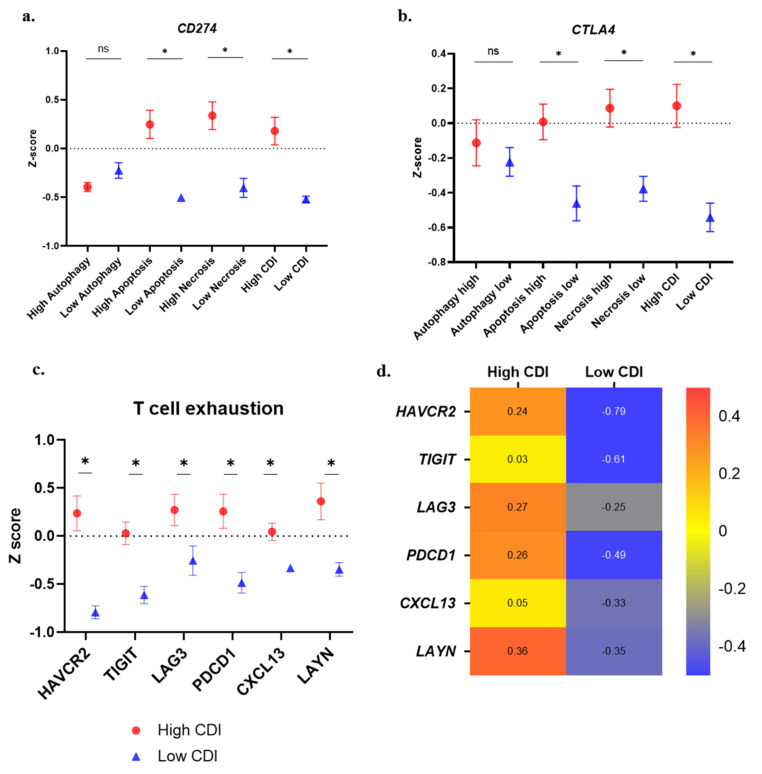
Immune suppressive features of patients with a high cell death index (CDI): (**a**) *CD274* gene expression; (**b**) *CTLA4* gene expression; (**c**) expression of T-cell exhaustion genes in patients with high CDI compared to lower CDI; and (**d**) the heatmap depicting the higher expression of T-cell exhaustion genes in high CDI group (median values) (* *p* < 0.05).

The expression of *CD274* and *CTLA4* was found to be higher in high apoptosis, necrosis, and CDI groups (Figure 6a,b). T cell exhaustion marker genes (*HAVCR2*, *TIGIT*, *LAG3*, *PDCD1*, *CXCL13*, *LAYN)* showed a higher expression in high autophagy, apoptosis, necrosis, and the CDI group (Figure 6c). The heatmap of all the median values reflected the higher expression of T cell exhaustion genes in the high-risk group (Figure 6d).

### 3.7. Enrichment Analysis

**Figure 7:**
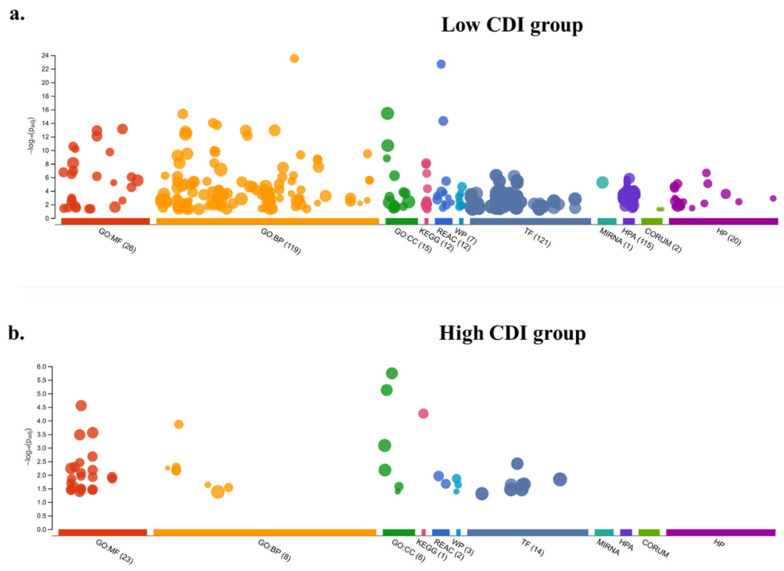
Functional enrichment analysis of highly expressed genes (log_2_ fold-change > 2) in the (**a**) low CDI group; and (**b**) high CDI group.

**Figure 8:**
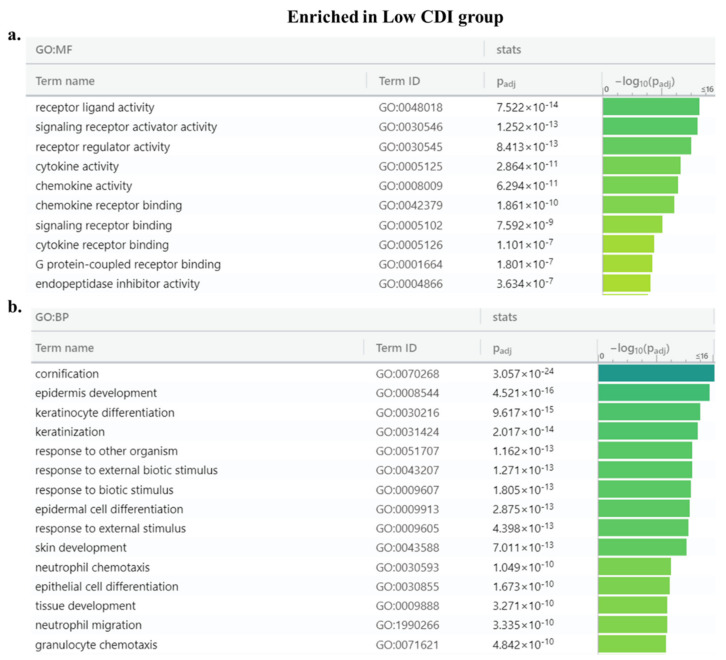

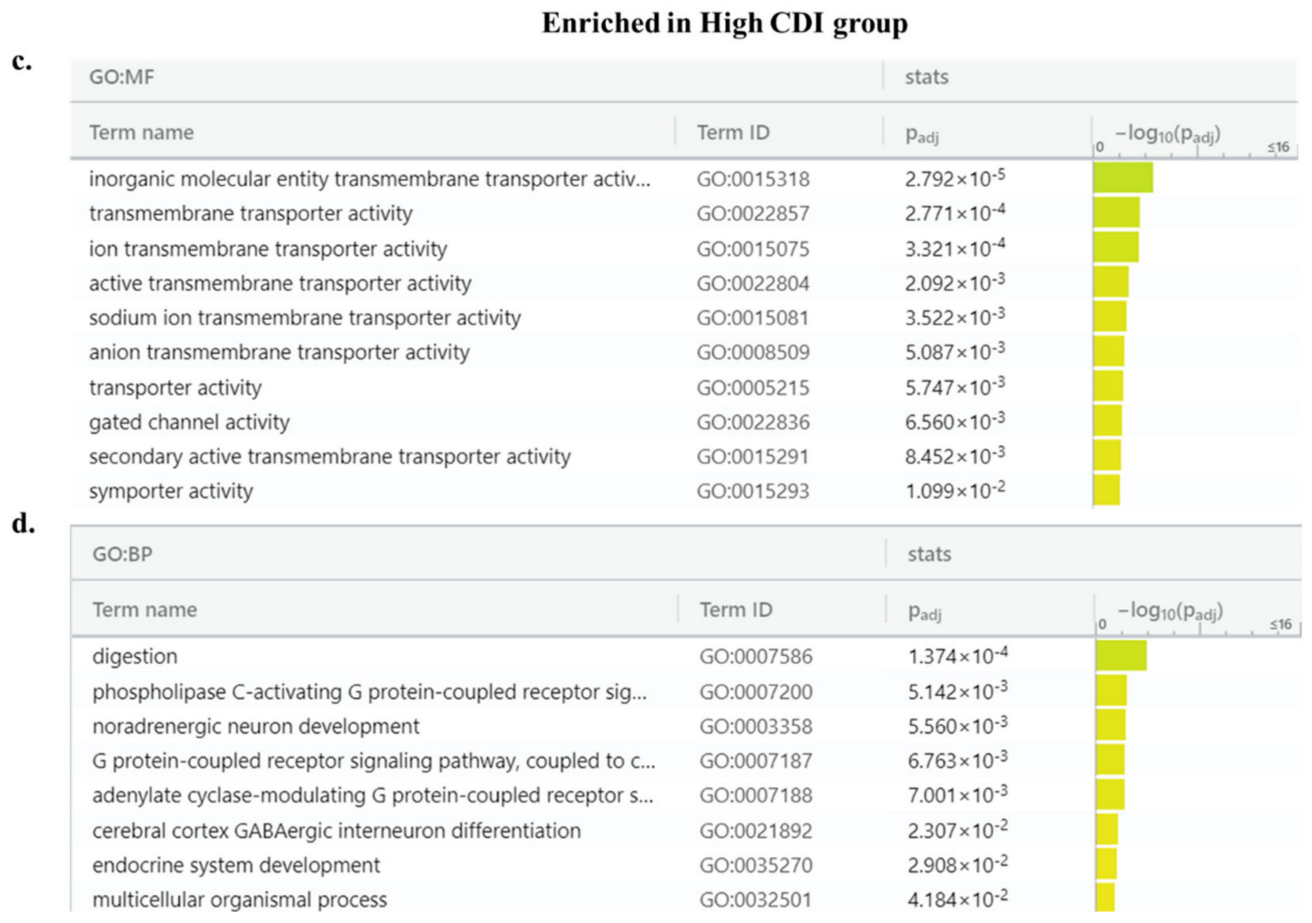
Pathways enriched in low and high CDI groups: (**a**,**b**) Molecular Function (GO: MF) and Biological Process (GO: BP) of the low CDI group and (**c**,**d**) the high CDI group.

Functional enrichment analysis of differential gene expression analysis between high and low CDI group revealed 943 genes with an inclusion filter of >2-fold for the differentially expressed genes. Among these, 329 genes were upregulated in high-risk patients while 614 genes were in the lower risk group (Figure 7a,b). The patients in the low CDI group had significant immune-related pathways whereas, the high CDI group lacked enrichment in immune pathways. The enriched Gene Ontology terms in low CDI groups were receptor ligand activity, signaling, cytokine and chemokine activity (Figure 8a,b). The enriched Gene Ontology terms in high CDI groups predominated by transmembrane transporters and gated channel activity (Figure 8c,d).

## 4. Discussion

Cell death is an essential component which plays a central role in the normal growth and development of an organism, however, its imbalance can result in several diseases including cancer [15]. Autophagy, apoptosis, and necrosis form three classically known cell death pathways. These pathways are active in the tumor microenvironment and can assist in tumor growth and metastasis [36]. Autophagy plays a dual role during the development and tumor progression. In the early stage, autophagy plays an anti-tumor role as it curtails inflammation while at the later stage, autophagy promotes tumorigenesis by fueling the energy and nutrient demands of cancer cells [37]. Furthermore, cancer cells show perturbation in the balance of pro-apoptotic and anti-apoptotic molecules affecting the apoptosis pathway [6]. Furthermore, tumor necrosis has been associated with the aggressive spread of cancer and reduced survival in lung cancer patients while apoptosis has shown mixed results [38]. In this study, we performed a prognostic evaluation of genes involved in cell-death pathways and its correlation with immune mediators and cells.

In the literature, there are several gene expression-based biomarkers which can stratify patients at high-risk which can be benefited from personalized therapy, but their accuracy and predictive potential remain limited [39,40,41]. However, most of these studies are the differential analysis of genomic or transcriptomics features which lack focus on biological pathways [22]. In this study, we explored the RNA-Seq data of LUAD patients for variations in cell death specific gene expression and its association with immune cells. We found that patients at high risk with a high cell death index (CDI) were associated with advanced stage and the involvement of the lymph node. This group also showed worse overall survival, progression-free survival and disease-specific survival. The positive correlation of CDI with clinicopathological parameters and survival outcome hints that the CDI is effective in the prognosis of LUAD patients. Further in this study, CD 8+ T cells, macrophages, and CAFs were found to be enriched in patients with high CDI. Neutrophils and endothelial cells were found to be enriched in patients with low CDI. Multiple studies have pointed to the fact that the tumor microenvironment can be modulated by tumors to propagate their own survival [42]. Even in the presence of T-cells, immunosuppressive molecules like CTLA4 or CD274 receptors can limit the responsive ability of T cells [43]. CTLA4 is present on the T cell surface and competes with the co-stimulatory receptor (CD28) present on T cells to bind to CD80/CD86 expressed by MHC-II present antigen-presenting cells (APCs) [44]. In our analysis, *CD274* and *CTLA4* were found to be expressed at a higher level in the high CDI patient group. CTLA4 has higher affinity compared to CD28 and thus can initiate a cascade of events which lead to the inhibition of the T cell response [45]. Moreover, T-regulatory cells constitutively express CTLA4 which further plays a critical role in the dampening of anti-tumor immunity [46]. In lung cancer, the infiltration of CD8+ T cells showed a higher expression of PD-1 and immunosuppressive functions [47]. The expression of CD274 has also been found to be upregulated in lung tumors and positively correlates with unfavorable prognosis [48,49]. Tumor-infiltrating lymphocytes (TILs), predominantly composed of CD8+ T cells have been found to be significantly associated with size, grade, vascular invasion and correlated with better clinical outcome [50]. However, these T cells can become dysfunctional or exhaustive, which is characterized by the high expression of inhibitory receptors. This exhaustive state results in the reduced production of immune response against cancer neoantigens and the impaired proliferation of these cells [51]. In our study, T cell exhaustion genes (*HAVCR2*, *TIGIT*, *LAG3*, *PDCD1*, *CXCL13* and *LYN)* showed higher expression in the high-risk group (high CDI). Furthermore, the pathway analysis identified a lower expression of diverse molecular and biological pathways in the high CDI patient group compared to patients in the low CDI group. As studied previously, variations in the functional immune network can lead to perturbations in anti-tumor response, immunoediting and the escape of cancer cells [52]. The tumor microenvironment biology is complex with features such as the inherent heterogeneity of tumors, variable density and the location of immune cells, temporal and spatial variations in the inflammatory and immunosuppressive response of same immune cells. This heterogeneity of the tumor immune microenvironment provides a unique opportunity to design targeted therapies [53]. In this study, the patients in the high CDI group characterized by the presence of T cell exhaustion genes and perturbed molecular and biological network provide a unique subset which can greatly benefit from checkpoint blockade immunotherapies.

## 5. Conclusions

In conclusion, this study identified patients at higher risk of mortality based on expression of cell-death based gene signature. Furthermore, in high-risk patients, the presence of the immunocompromised microenvironment indicated by the higher infiltration of cytotoxic T cells along with the presence of checkpoint molecules and T cell exhaustion genes presents a unique subgroup which could be targeted by checkpoint inhibitors. These patients at higher risk might be more suitable to benefit from the PD-1/PD-L1 or CTLA-4 blockade. Further evaluation of these findings using a prospective cohort is essential to assess the validity of these gene signatures. With further validation, this clinical signature can be explored as prognostic and predictive biomarker panel to design personalized therapies for LUAD patients.

## Figures and Tables

**Table 1:** The prognostically significant gene signature within each category of cell death in lung adenocarcinoma.

Cell Death Process	Gene	Gene ID	Gene (Full Name)
Autophagy	*PRKAA2*	5563	Protein Kinase AMP-Activated Catalytic Subunit Alpha 2
	*ATG12*	9140	Autophagy Related 12
	*ULK2*	9706	Unc-51 Like Autophagy Activating Kinase 2
	*ATG5*	9474	Autophagy Related 5
	*GABARAPL1*	23710	GABA Type A Receptor Associated Protein Like 1
Apoptosis	*BCL2L1*	598	B-Cell Lymphoma 2 Like 1
	*CASP9*	842	Caspase 9
	*CYCS*	54205	C, Somatic
	*IL1A*	3552	Interleukin 1 Alpha
	*PIK3CG*	5294	Phosphatidylinositol-4,5-Bisphosphate 3-Kinase Catalytic Subunit Gamma
	*TNFRSF10D*	8793	Tumor Necrosis Factor Receptor Superfamily Member 10D
	*FADD*	8772	Fas Associated Via Death Domain
Apoptosis and Necrosis	*BIRC3*	330	Baculoviral IAP Repeat-Containing Protein 3
	*FAS*	355	Fas Cell Surface Death Receptor
Necrosis	*DNM1L*	10059	Dynamin 1 Like
	*GSDME*	1687	Gasdermin E
	*IPMK*	253430	Inositol Polyphosphate Multikinase
	*MLKL*	197259	Mixed Lineage Kinase Domain Like Pseudokinase
	*RBCK1*	10616	RANBP2-Type and C3HC4-Type Zinc Finger Containing 1
	*TICAM1*	148022	Toll Like Receptor Adaptor Molecule 1
	*YBX3*	8531	Y-Box Binding Protein 3

**Table 2:** (**a**) Comparison of the clinico-pathological features of patients with high CDI (*n* = 301 patients) and low CDI (*n* = 209 patients) using Pearson’s chi-square analysis.(**b**) Univariate and multivariate cox regression analysis of clinicopathological variables associated with the cell death index (CDI). Statistically significant values are shown in bold.

(a)							
Clinical Variable		High CDI (*n* = 301)		Low CDI (*n* = 209)		Pearson χ^2^ *p*-Value	
**Age**							
<66 years		137		101		0.63	
>66 years		151		102			
**Ethnicity**							
African American		29		23		0.72	
Caucasian		224		160			
**Sex**							
Female		154		120		0.16	
Male		147		89			
**Stage**							
I + II		225		173		**0.01**	
II + III		76		34			
**Lymph Node Involvement**							
N0		174		154		**0.01**	
N1 + N2 + N3		122		49			
**Distant Metastasis**							
No Metastasis M0		214		127		0.78	
Metastasis M1		15		10			
**Aneuploidy Score**							
<16		127		114		**0.01**	
>16		166		90			
**Fraction Genome Altered**							
<0.23		135		107		0.22	
>0.23		156		99			
**Overall Survival (OS)**							
<5 years		267		177		0.06	
>5 years		25		28			
**OS Status**							
Living		174		151		**0.01**	
Deceased		127		58			
**Progression-Free Survival (PFS)**							
<5 years		277		186		0.06	
>5 years		17		21			
**PFS Status**							
No progression		165		139		**0.01**	
Progression		136		70			
**Disease-Free Survival (DFS)**							
<5 Years		139		121		0.39	
>5 Years		17		20			
**DFS Status**							
Disease-free		102		111		**0.01**	
Recurred/progressed		56		31			
**Disease-Specific Survival (DSS)**							
<5 years		267		177		0.06	
>5 years		25		28			
**DSS Status**							
Tumor free		194		167		**0.01**	
Dead with tumor		84		29			
**(b)**							
**Clinical Variable**	**Univariate**			**Multivariate**			
	**Hazard Ratio**	**95% CI**	***p*-Value**	**Hazard Ratio**	**95% CI**		***p*-Value**
Cell death index (CDI High, CDI low)	1.75	1.28–2.45	**<0.001**	1.62	1.11–2.36		**<0.01**
T, Stage (III + IV, I + II)	2.68	1.95–3.63	**<0.001**	2.13	1.39–3.25		**<0.001**
N, Lymph Node involvement (N1 + N2 + N3, N0)	2.61	1.94–3.52	**<0.001**	2.25	1.59–3.19		**<0.001**
M, Distant Metastasis (M1, M0)	2.12	1.19–3.52	**<0.01**	1.68	0.93–3.03		0.07
Age (>66, <66 years)	1.2	0.89–1.62	0.21				
Sex (Male, Female)	1.05	0.71–0.78	0.71				
Radiation therapy (Yes, No)	2.02	1.36–2.90	**<0.001**				

## Data Availability

The study utilized datasets from The Cancer Genome Atlas Program and are freely available to public.

## Supplementary Materials

The following are available online at https://www.mdpi.com/2072-6694/13/1/155/s1, Table S1: Prognostic analysis (Log-rank p-values) of Autophagy related genes in LUAD patients, Table S2: Prognostic analysis (Log-rank p-values) of Apoptosis related genes in LUAD patients, Table S3: Prognostic analysis (Log-rank p-values) of Necrosis related genes in LUAD patients, Table S4: Upregulated (329 genes) expressed at >2-fold in high risk group, Table S5: Upregulated (614 genes) expressed at >2-fold in low risk group, Table S6: A list of cytokines in pathway: ‘KEGG cytokine-cytokine receptor interaction’ (*n* = 265 genes) analyzed in this study.
