# Supplementary material for: Immunogenomic Gene Signature of Cell-Death Associated Genes with Prognostic Implications in Lung Cancer
Source: Cancers (Basel). 2021 Jan 5;13(1):155. doi: 10.3390/cancers13010155 (PMC7795632; doi:10.3390/cancers13010155)
Supplement: Supplementary file 1 [file cancers-13-00155-s001.pdf]

cancers

MDPI

Supplementary Material

Table S1. Prognostic analysis (Log-rank p-values) of Autophagy related genes in LUAD patients.

GENE	OS	DFS	PFS	DSS
ATG12	0.14	0.38	0.38	0.01*
ATG3	0.38	0.45	0.72	0.35
ATG4A	0.09	0.31	0.85	0.16
ATG4B	0.76	0.48	0.58	0.73
ATG4C	0.31	0.32	0.72	0.65
ATG4D	0.63	0.67	0.96	0.63
ATG5	0.25	0.55	0.46	0.82
ATG7	0.6	0.7	0.05	0.31
BECN1	0.71	0.62	0.59	0.75
BECN2	NA	NA	NA	NA
GABARAP	0.54	0.88	0.3	0.25
GABARAPL1	0.001*	0.27	0.001*	0.001*
GABARAPL2	0.71	0.35	0.08	0.71
IFNA1	0.12	0.95	0.53	0.07
IFNA10	0.27	0.87	0.53	0.36
IFNA13	0.08	0.38	0.58	0.13
IFNA14	0.62	0.64	0.32	0.56
IFNA16	0.31	0.06	0.44	0.26
IFNA17	0.48	0.01*	0.11	0.57
IFNA2	0.57	0.46	0.28	0.98
IFNA21	0.65	0.72	0.89	0.63
IFNA4	0.18	0.91	0.12	0.18
IFNA5	0.55	0.35	0.47	0.96
IFNA6	NA	NA	NA	NA
IFNA7	NA	NA	NA	NA
IFNA8	0.01*	0.58	0.01*	0.01*
IFNG	0.39	0.24	0.1	0.58
INS	0.45	0.52	0.4	NA
PIK3C3	0.73	0.45	0.69	0.44
PIK3R4	0.88	0.45	0.31	0.68
PRKAA1	0.61	0.64	0.65	0.69
PRKAA2	0.37	0.22	0.34	0.3
ULK1	0.81	0.52	0.84	0.55
ULK2	0.74	0.22	0.06	0.93

Table S2. Prognostic analysis (Log-rank p-values) of Apoptosis related genes in LUAD patients.

Gene	OS	DFS	PFS	DSS
AIFM1	0.93	0.64	0.18	0.44
AKT1	0.93	0.52	0.88	0.95
AKT2	0.77	0.71	0.22	0.23
AKT3	0.1	0.29	0.61	0.58
APAF1	0.29	0.64	0.4	0.08
ATM	0.44	0.96	0.58	0.53
BAD	0.41	0.25	0.62	0.79
BAX	0.02*	0.75	0.19	0.05*
BCL2	0.56	0.19	0.23	0.99
BCL2L1	0.01*	0.01*	0.01*	0.01*
BID	0.69	0.78	0.8	0.39
BIRC2	0.15	0.17	0.12	0.14
BIRC3	0.01*	0.68	0.01*	0.01*
CAPN1	0.59	0.22	0.41	0.06
CAPN2	0.48	0.4	0.92	0.61
CASP10	0.19	0.33	0.44	0.89

CASP3	0.85	0.93	0.77	0.86
CASP6	0.43	0.26	0.42	0.34
CASP7	0.95	0.74	0.37	0.41
CASP8	0.67	0.31	0.06	0.27
CASP9	0.01*	0.94	0.01*	0.01*
CFLAR	0.77	0.47	0.67	0.78
CHP1	0.65	0.19	0.84	0.47
CHP2	0.01*	0.16	0.41	0.05*
CHUK	0.25	0.39	0.61	0.31
CSF2RB	0.11	0.15	0.49	0.35
CYCS	0.04*	0.68	0.03*	0.04*
DFFA	0.84	0.98	0.5	0.31
DFFB	0.96	0.33	0.54	0.63
ENDOD1	0.33	0.49	0.89	0.28
ENDOG	0.75	0.95	0.93	0.52
EXOG	0.41	0.05*	0.02*	0.35
FADD	0.1	0.49	0.03*	0.01*
FAS	0.05*	0.05*	0.06	0.01*
FASLG	0.1	0.52	0.93	0.13
IKBKB	0.58	0.96	0.44	0.33
IKBKG	0.07	0.36	0.5	0.11
IL1A	0.01*	0.01*	0.01*	0.06
IL1B	0.22	0.38	0.3	0.29
IL1R1	0.3	0.3	0.01*	0.4
IL1RAP	0.09	0.01*	0.3	0.58
IL3	0.64	0.79	0.98	0.34
IL3RA	0.01*	0.1	0.32	0.07
IRAK1	0.87	0.62	0.77	0.42
IRAK2	0.82	0.81	0.58	0.52
IRAK3	0.94	0.7	0.92	0.84
MAP3K14	0.84	0.34	0.58	0.54
MYD88	0.45	0.19	0.59	0.56
NFKB1	0.54	0.98	0.26	0.78
NFKBIA	0.31	0.06	0.02*	0.72
NGF	0.38	0.54	0.83	0.53
NTRK1	0.45	0.06	0.38	0.12
PIK3CA	0.02*	0.3	0.1	0.29
PIK3CB	0.54	0.45	0.99	0.6
PIK3CD	0.44	0.01*	0.07	0.63
PIK3CG	0.01*	0.03*	0.04*	0.03*
PIK3R1	0.26	0.17	0.03*	0.43
PIK3R2	0.03*	0.46	0.62	0.13
PIK3R3	0.64	0.68	0.67	0.73
PIK3R5	0.09	0.34	0.75	0.1
PPP3CA	0.27	0.1	0.73	0.79
PPP3CB	0.69	0.37	0.83	0.83
PPP3CC	0.86	0.92	0.24	0.34
PPP3R1	0.76	0.64	0.93	0.51
PPP3R2	0.65	0.53	0.99	0.17
PRKACA	0.6	0.5	0.69	0.73
PRKACB	0.94	0.28	0.56	0.83
PRKACG	0.97	0.96	0.75	0.84
PRKAR1A	0.41	0.93	0.21	0.41
PRKAR1B	0.04*	0.45	0.17	0.09
PRKAR2A	0.93	0.21	0.18	0.78
PRKAR2B	0.79	0.72	0.84	0.69
PRKX	0.41	0.43	0.73	0.58
RELA	0.17	0.62	0.2	0.15
RIPK1	0.34	0.64	0.29	0.26
TNF	0.44	0.68	0.32	0.71
TNFRSF10A	0.93	0.02*	0.25	0.64
TNFRSF10B	0.01*	0.11	0.19	0.09
TNFRSF10C	0.07	0.73	0.35	0.03*

TNFRSF10D	0.03*	0.04*	0.01*	0.11
TNFRSF1A	0.01*	0.98	0.01*	0.05*
TNFSF10	0.79	0.4	0.79	0.71
TP53	0.7	0.24	0.92	0.48
TRADD	0.68	0.11	0.47	0.76
TRAF2	0.35	0.73	0.82	0.99
XIAP	0.9	0.07	0.18	0.95

Table S3. Prognostic analysis (Log-rank p-values) of Necrosis related genes in LUAD patients.

Gene	OS	DFS	PFS	DSS
BAX	0.02*	0.75	0.19	0.05*
BIRC2	0.15	0.17	0.12	0.14
BIRC3	0.01*	0.68	0.01*	0.01*
CASP8	0.67	0.31	0.06	0.27
CFLAR	0.77	0.47	0.67	0.78
FADD	0.1	0.49	0.03*	0.01*
FAS	0.05*	0.05*	0.06	0.01*
FASLG	0.1	0.52	0.93	0.13
RIPK1	0.34	0.64	0.29	0.26
TNF	0.44	0.68	0.32	0.71
TP53	0.7	0.24	0.92	0.48
TRAF2	0.35	0.73	0.82	0.99
ALKBH7	0.72	0.14	0.08	0.58
ARHGEF2	0.74	0.58	0.25	0.33
BNIP3	0.18	0.9	0.15	0.18
BOK	0.45	0.83	0.72	0.36
CAV1	0.35	0.84	0.22	0.12
CD14	0.19	0.28	0.62	0.28
CYLD	0.89	0.34	0.7	0.82
DNM1L	0.04*	0.07	0.03*	0.01*
FZD9	0.78	0.73	0.79	0.69
GSDME	0.04*	0.01*	0.01*	0.01*
HEBP2	0.54	0.32	0.28	0.91
IPMK	0.01*	0.53	0.22	0.01*
IRF3	0.39	0.28	0.31	0.39
ITPK1	0.16	0.23	0.93	0.63
LY96	0.46	0.21	0.75	0.83
MAP3K5	0.52	0.84	0.49	0.38
MLKL	0.01*	0.55	0.44	0.48
MT-CO2	NA	NA	NA	NA
MT3	0.14	0.69	0.94	0.43
MTCO2P12	NA	NA	NA	NA
PELI1	0.81	0.95	0.62	0.84
PGAM5	0.56	0.26	0.29	0.53
PPIF	0.3	0.54	0.87	0.68
PYGL	0.13	0.94	0.81	0.99
RBCK1	0.01*	0.42	0.31	0.07
RIPK3	0.79	0.6	0.19	0.68
SLC25A4	0.86	0.93	0.3	0.98
SPATA2	0.89	0.78	0.27	0.75
TICAM1	0.01*	0.33	0.28	0.01*
TICAM2	0.63	0.65	0.5	0.29
TLR3	0.85	0.47	0.58	0.48
TLR4	0.09	0.4	0.51	0.13
TMEM123	0.75	0.57	0.89	0.48
TRPM7	0.89	0.48	0.7	0.42
TSPO	0.43	0.73	0.72	0.4
UCN	0.65	0.55	0.72	0.68
YBX3	0.01*	0.64	0.18	0.13

Table S4. Upregulated (329 genes) expressed at > 2-fold in high risk group.

GENE ID	*Gene*	baseMean	log2FoldChange	lfcSE	stat	pvalue	padj
309	*SLC10A2*	131.7256	8.871153	1.063901	8.338325	7.54E-17	0.00
18	*PCSK2*	3736.282	8.631713	0.686807	12.56788	3.17E-36	0.00
20	*SLC14A2*	114.2138	7.251858	0.58867	12.31905	7.15E-35	0.00
23	*CELF3*	53.52999	7.182198	0.589741	12.17855	4.04E-34	0.00
47	*PGC*	42949.43	6.565064	0.588595	11.15379	6.86E-29	0.00
44	*TAC4*	21.78908	5.867511	0.520617	11.27031	1.84E-29	0.00
965	*PAX7*	203.3074	5.788932	0.871478	6.642664	3.08E-11	0.00
444	*SLC13A2*	76.24164	5.432022	0.691706	7.853081	4.06E-15	0.00
273	*ASCL1*	196.4388	5.378043	0.633881	8.484315	2.17E-17	0.00
588	*CALCA*	394.8732	5.250791	0.709025	7.405651	1.31E-13	0.00
2386	*WFDC5*	34.02593	5.061172	0.987889	5.123219	3.00E-07	0.00
809	*C1QL2*	38.51167	4.929595	0.71082	6.935086	4.06E-12	0.00
252	*GKN2*	602.6402	4.904697	0.569033	8.619354	6.73E-18	0.00
687	*TMEM229A*	13.35952	4.669075	0.651362	7.168169	7.60E-13	0.00
1028	*CEACAM8*	7.650484	4.660928	0.713214	6.535108	6.36E-11	0.00
75	*CNGA3*	81.45622	4.61744	0.448175	10.30276	6.85E-25	0.00
96	*UMODL1*	102.7945	4.389354	0.438582	10.00806	1.40E-23	0.00
912	*BPIL1*	104.8604	4.327812	0.644153	6.718609	1.83E-11	0.00
219	*SLC1A7*	697.6253	4.324529	0.486707	8.885274	6.38E-19	0.00
4620	*SOX14*	2.065215	4.300192	1.090805	3.942219	8.07E-05	0.00
362	*HCN1*	18.51129	4.287165	0.52606	8.149582	3.65E-16	0.00
518	*CRHR2*	12.91158	4.240173	0.555276	7.636159	2.24E-14	0.00
281	*CHIA*	395.3987	4.213058	0.498446	8.452393	2.85E-17	0.00
31	*TMEM130*	601.6394	4.168308	0.352113	11.83799	2.48E-32	0.00
592	*CDC20B*	43.65202	4.107341	0.555904	7.388585	1.48E-13	0.00
290	*PLA2G1B*	115.9732	4.066262	0.483452	8.410893	4.07E-17	0.00
1673	*GRK1*	3.662612	3.982382	0.69209	5.754136	8.71E-09	0.00
312	*MEGF11*	67.39387	3.974526	0.477818	8.318085	8.94E-17	0.00
54	*NOTUM*	201.7405	3.92414	0.362408	10.82796	2.54E-27	0.00
190	*KLB*	201.9029	3.8806	0.425955	9.110346	8.21E-20	0.00
378	*C1orf95*	81.67584	3.86277	0.477561	8.088532	6.04E-16	0.00
4196	*GP2*	4.920758	3.857883	0.933959	4.130676	3.62E-05	0.00
311	*SCGB3A1*	4697.08	3.85109	0.462949	8.318603	8.90E-17	0.00
257	*GFRA3*	491.6787	3.835159	0.447215	8.575651	9.85E-18	0.00
491	*AMBP*	196.0312	3.833322	0.496259	7.724444	1.12E-14	0.00
2174	*CA6*	3.158708	3.805081	0.720513	5.281074	1.28E-07	0.00
383	*PRMT8*	26.08747	3.781157	0.468424	8.072076	6.91E-16	0.00
1270	*HMGCS2*	31.55951	3.771948	0.607872	6.205169	5.46E-10	0.00
896	*UCN3*	45.07848	3.750302	0.555005	6.757235	1.41E-11	0.00
1578	*NT5C1A*	2.676489	3.707608	0.634382	5.844439	5.08E-09	0.00
4624	*DLK1*	22.63856	3.700166	0.938965	3.940687	8.12E-05	0.00
241	*TMEM59L*	454.5112	3.684886	0.422868	8.714027	2.93E-18	0.00
204	*HSD17B13*	73.81107	3.682636	0.409527	8.992415	2.42E-19	0.00
213	*NUP210L*	43.09121	3.675772	0.411685	8.928594	4.31E-19	0.00
42	*KIAA1324*	2467.197	3.674156	0.32232	11.3991	4.22E-30	0.00
3361	*CALCB*	6.267565	3.65409	0.806221	4.53237	5.83E-06	0.00
2681	*ASZ1*	3.198188	3.653016	0.740737	4.931599	8.16E-07	0.00
40	*SYT2*	106.6527	3.626768	0.315323	11.50176	1.29E-30	0.00
7875	*LGI1*	1.144995	3.599798	1.334231	2.698032	0.006975	0.02
1997	*FADS6*	19.02862	3.590361	0.662489	5.419504	5.98E-08	0.00
1470	*LOC729668*	5.886467	3.582718	0.599872	5.972475	2.34E-09	0.00
1379	*COL25A1*	144.7839	3.548253	0.583887	6.076955	1.22E-09	0.00
89	*ENO3*	299.7375	3.538559	0.351464	10.06806	7.65E-24	0.00
1545	*CASR*	15.76484	3.537764	0.602259	5.874157	4.25E-09	0.00
2013	*GLB1L3*	259.9133	3.532241	0.653255	5.407137	6.40E-08	0.00
5777	*NEUROD1*	4.991265	3.494238	1.011082	3.455939	0.000548	0.00
9046	*SSX5*	1.303196	3.494046	1.49931	2.330436	0.019783	0.04
937	*INSM1*	12.83102	3.477523	0.520424	6.682098	2.36E-11	0.00
1367	*CCDC129*	32.7197	3.470739	0.569942	6.089637	1.13E-09	0.00
887	*FAM166A*	2.343638	3.451419	0.509725	6.771137	1.28E-11	0.00

6292	*DMRTC1*	3.918784	3.429365	1.056605	3.245644	0.001172	0.00
610	*FBN3*	68.79615	3.429002	0.466733	7.346817	2.03E-13	0.00
500	*SCGB3A2*	9450.273	3.371222	0.437967	7.69743	1.39E-14	0.00
710	*LHFPL3*	26.411	3.365006	0.47273	7.118241	1.09E-12	0.00
3227	*NELL1*	170.0442	3.339552	0.723795	4.613949	3.95E-06	0.00
1891	*LHCGR*	4.151728	3.319584	0.601091	5.522597	3.34E-08	0.00
884	*LOC400794*	7.926637	3.309334	0.488564	6.773594	1.26E-11	0.00
7503	*EIF4E1B*	3.426779	3.308789	1.175944	2.813729	0.004897	0.01
2056	*RGS7*	16.96028	3.29808	0.613384	5.376858	7.58E-08	0.00
4663	*POU3F2*	64.48936	3.29031	0.838258	3.925175	8.67E-05	0.00
921	*LOC145837*	161.4876	3.283242	0.489054	6.713454	1.90E-11	0.00
70	*SUSD2*	4231.595	3.282468	0.31499	10.42088	1.99E-25	0.00
775	*LOC723809*	66.15443	3.247309	0.464017	6.998259	2.59E-12	0.00
793	*ODZ1*	231.6695	3.241721	0.465637	6.9619	3.36E-12	0.00
464	*C9orf173*	10.2713	3.237753	0.414821	7.805185	5.94E-15	0.00
1388	*FRMPD2*	6.769056	3.229939	0.532307	6.067811	1.30E-09	0.00
1412	*WIF1*	872.1761	3.221695	0.533217	6.041994	1.52E-09	0.00
6701	*BASE*	1.028466	3.193225	1.033603	3.089411	0.002006	0.01
509	*SLC26A5*	9.190664	3.181778	0.414707	7.672355	1.69E-14	0.00
113	*SCUBE3*	198.5567	3.175152	0.323761	9.807098	1.05E-22	0.00
5379	*LOC116437*	2.527163	3.169904	0.87692	3.614815	0.000301	0.00
4382	*TFAP2D*	8.56581	3.16609	0.782645	4.045372	5.22E-05	0.00
557	*SERPIND1*	195.9107	3.165382	0.422136	7.498486	6.46E-14	0.00
122	*SCUBE2*	426.7389	3.162877	0.326765	9.679351	3.69E-22	0.00
2095	*C15orf50*	1.585375	3.154259	0.58978	5.348198	8.88E-08	0.00
106	*CHAD*	57.76026	3.141839	0.317339	9.900563	4.14E-23	0.00
3933	*SLC17A3*	1.562911	3.129837	0.735756	4.253907	2.10E-05	0.00
266	*CAC2D2*	1171.054	3.121269	0.366254	8.522149	1.57E-17	0.00
1555	*GALNTL6*	12.96144	3.111999	0.530502	5.866138	4.46E-09	0.00
6801	*ISX*	10.45373	3.101684	1.017411	3.048606	0.002299	0.01
1581	*RIMBP2*	68.1186	3.069899	0.52538	5.843203	5.12E-09	0.00
224	*CYP2B7P1*	2750.809	3.066363	0.347355	8.827763	1.07E-18	0.00
140	*TDRD10*	90.94129	3.042657	0.320684	9.488035	2.35E-21	0.00
9394	*ADAM2*	1.323909	3.034539	1.365176	2.222819	0.026228	0.05
528	*C16orf89*	5175.62	3.027483	0.39864	7.594535	3.09E-14	0.00
478	*PLA2G10*	468.0149	3.01619	0.387818	7.777334	7.41E-15	0.00
566	*PCP4L1*	477.7283	2.986249	0.40027	7.460584	8.61E-14	0.00
469	*LRRC26*	15.14819	2.984353	0.382718	7.79778	6.30E-15	0.00
5714	*LECT1*	4.087393	2.975101	0.854824	3.480366	0.000501	0.00
5957	*GOLGA6L1*	1.372918	2.972253	0.878729	3.382445	0.000718	0.00
798	*SPINK5*	597.0724	2.955642	0.425255	6.950286	3.65E-12	0.00
5241	*C10orf71*	1.100513	2.923856	0.796875	3.669151	0.000243	0.00
795	*GPC5*	35.01635	2.895727	0.416119	6.958884	3.43E-12	0.00
1149	*CRLF1*	2311.304	2.894491	0.45445	6.369224	1.90E-10	0.00
1691	*CYP17A1*	7.492115	2.89173	0.503577	5.742379	9.34E-09	0.00
634	*BRSK2*	40.65598	2.874446	0.39482	7.280399	3.33E-13	0.00
1275	*KIAA1210*	3.18128	2.870406	0.462951	6.200239	5.64E-10	0.00
7337	*STRA8*	1.145056	2.861391	0.997424	2.86878	0.004121	0.01
158	*FNDC5*	75.24044	2.859055	0.304901	9.377006	6.79E-21	0.00
1708	*TEPP*	23.60669	2.8551	0.499099	5.720505	1.06E-08	0.00
4870	*XAGE2*	55.11452	2.843639	0.741259	3.836227	0.000125	0.00
4633	*CYP1A2*	1.354497	2.84205	0.721697	3.938007	8.22E-05	0.00
8177	*TBL1Y*	1.048638	2.841556	1.091479	2.603399	0.00923	0.02
2928	*LRFN2*	1.97328	2.841321	0.594494	4.779397	1.76E-06	0.00
3653	*ASPG*	70.95969	2.8272	0.644466	4.386888	1.15E-05	0.00
330	*FLJ42875*	36.99397	2.826551	0.342921	8.242574	1.69E-16	0.00
472	*FZD9*	52.05953	2.819076	0.362082	7.785742	6.93E-15	0.00
5367	*SLC38A8*	1.152451	2.813607	0.777181	3.620272	0.000294	0.00
382	*LEFTY2*	22.81246	2.790037	0.345358	8.078678	6.55E-16	0.00
2718	*ABCC8*	9.674117	2.786769	0.568203	4.90453	9.37E-07	0.00
1492	*PCP4*	82.66852	2.77808	0.46827	5.932645	2.98E-09	0.00
1036	*NPAS3*	84.80157	2.77288	0.425015	6.524186	6.84E-11	0.00
3243	*HTR4*	1.611574	2.769694	0.601616	4.603758	4.15E-06	0.00

175	*CLIC6*	2845.045	2.766789	0.298927	9.255725	2.13E-20	0.00
639	*ADRA2A*	440.1548	2.766754	0.380607	7.269325	3.61E-13	0.00
335	*SRPK3*	86.96155	2.76439	0.335779	8.232764	1.83E-16	0.00
8082	*SOX1*	2.589366	2.759595	1.048782	2.631237	0.008507	0.02
512	*PTGER3*	83.34211	2.752142	0.359423	7.657107	1.90E-14	0.00
3872	*ALB*	50.35954	2.745919	0.641464	4.280707	1.86E-05	0.00
5194	*DJC5G*	0.984118	2.745209	0.744265	3.688483	0.000226	0.00
283	*PRDM16*	321.1088	2.722754	0.322307	8.447708	2.97E-17	0.00
2306	*MS4A15*	453.6124	2.719662	0.524963	5.180677	2.21E-07	0.00
714	*KCNH2*	249.3575	2.691478	0.378522	7.110493	1.16E-12	0.00
977	*C1orf65*	8.106462	2.686294	0.406094	6.614951	3.72E-11	0.00
10907	*GC*	0.917147	2.683581	1.485196	1.806886	0.07078	0.12
118	*IRX5*	345.2835	2.68001	0.274775	9.753475	1.78E-22	0.00
3628	*C13orf35*	1.365064	2.674155	0.607622	4.401018	1.08E-05	0.00
490	*LPL*	1507.559	2.673245	0.345713	7.732554	1.05E-14	0.00
537	*CLCNKA*	16.59118	2.6608	0.3514	7.571994	3.68E-14	0.00
6381	*C19orf41*	5.867751	2.652852	0.826489	3.209783	0.001328	0.00
3642	*COL2A1*	16.03196	2.652267	0.603897	4.391918	1.12E-05	0.00
1212	*TAS1R1*	15.47478	2.651866	0.421704	6.288456	3.21E-10	0.00
51	*CIT*	2982.19	2.638245	0.241768	10.91232	1.01E-27	0.00
6861	*SLC17A1*	0.719624	2.637106	0.871168	3.027093	0.002469	0.01
2722	*SLC7A10*	91.2015	2.63456	0.537707	4.899623	9.60E-07	0.00
442	*GPR98*	439.7709	2.633484	0.335161	7.857366	3.92E-15	0.00
331	*KIAA1984*	26.21864	2.624148	0.318457	8.240209	1.72E-16	0.00
8956	*MCCD1*	2.186491	2.621784	1.112078	2.357554	0.018396	0.04
170	*CPAMD8*	633.0195	2.615623	0.281959	9.276619	1.75E-20	0.00
376	*IGFALS*	52.17811	2.614583	0.322899	8.097209	5.62E-16	0.00
10860	*SLC6A18*	0.649413	2.613442	1.435843	1.820145	0.068737	0.12
3234	*TRIM63*	4.462879	2.60886	0.565871	4.610344	4.02E-06	0.00
6564	*SOX3*	1.093836	2.599079	0.826828	3.143435	0.00167	0.00
1299	*CYP2A6*	6.042634	2.595586	0.420113	6.178298	6.48E-10	0.00
888	*ACE2*	362.2851	2.589395	0.382496	6.769725	1.29E-11	0.00
3867	*WFDC12*	7.566326	2.584374	0.603487	4.282403	1.85E-05	0.00
52	*SELENBP1*	4738.253	2.577193	0.236477	10.8983	1.17E-27	0.00
11489	*TRIM48*	0.573959	2.568258	1.547316	1.659815	0.096952	0.15
10829	*CCKAR*	0.819296	2.566022	1.400811	1.831812	0.066979	0.11
4176	*TRIM71*	5.004219	2.551412	0.616015	4.141799	3.45E-05	0.00
984	*TMEM63C*	206.2274	2.536708	0.384544	6.596662	4.21E-11	0.00
143	*SHE*	643.4181	2.532886	0.267287	9.476262	2.64E-21	0.00
1815	*PIGR*	17629.08	2.528023	0.451076	5.604426	2.09E-08	0.00
732	*SFTA3*	3040.387	2.512779	0.354675	7.084746	1.39E-12	0.00
1386	*SLC38A3*	15.12138	2.507927	0.41326	6.068639	1.29E-09	0.00
765	*SLC30A3*	19.50776	2.497601	0.355685	7.021952	2.19E-12	0.00
3566	*MSMB*	262.8568	2.49721	0.563783	4.42938	9.45E-06	0.00
10287	*LOC100190940*	24.25829	2.49575	1.262728	1.976476	0.048101	0.09
3031	*FAM182A*	2.659614	2.494546	0.527508	4.728922	2.26E-06	0.00
11602	*PIWIL3*	1.734635	2.494353	1.526631	1.633894	0.102281	0.16
3454	*SLC6A3*	144.13	2.491594	0.555442	4.485784	7.26E-06	0.00
521	*CDH15*	115.1314	2.489771	0.326295	7.630426	2.34E-14	0.00
1040	*PLEKHG4B*	336.7386	2.483469	0.381276	6.513564	7.34E-11	0.00
1556	*UPK3A*	24.04053	2.482517	0.423303	5.864638	4.50E-09	0.00
2185	*MYBPHL*	52.78452	2.481183	0.470489	5.273629	1.34E-07	0.00
298	*FAM182B*	32.19593	2.480355	0.296111	8.37643	5.46E-17	0.00
206	*GNMT*	13.35458	2.475864	0.276268	8.961831	3.19E-19	0.00
5605	*FRMD1*	1.124118	2.46083	0.696875	3.531237	0.000414	0.00
5759	*ZSCAN10*	0.893583	2.452404	0.708273	3.462512	0.000535	0.00
3168	*ANKRD34B*	59.0995	2.440575	0.524758	4.65086	3.31E-06	0.00
2640	*ARX*	77.06872	2.431494	0.490082	4.961404	7.00E-07	0.00
7023	*BRDT*	127.0921	2.428124	0.817338	2.970771	0.002971	0.01
1801	*SLC5A2*	8.449396	2.427806	0.432437	5.614247	1.97E-08	0.00
2396	*RLN3*	1.999725	2.420487	0.473098	5.116251	3.12E-07	0.00
554	*KCNE4*	519.6581	2.416777	0.322008	7.505341	6.13E-14	0.00
1541	*C8orf85*	54.44046	2.416754	0.411	5.88018	4.10E-09	0.00

669	*ADRB1*	78.81826	2.409599	0.334661	7.20011	6.02E-13	0.00
3219	*APOH*	150.0528	2.408025	0.521694	4.615776	3.92E-06	0.00
2703	*LRRC14B*	1.398955	2.406549	0.489589	4.915444	8.86E-07	0.00
3747	*TMEM132D*	48.44695	2.401705	0.55366	4.337867	1.44E-05	0.00
4514	*LOC150622*	10.86422	2.395565	0.601336	3.983735	6.78E-05	0.00
2028	*ATP4B*	2.556559	2.386831	0.442252	5.396995	6.78E-08	0.00
1677	*SLC26A9*	1507.456	2.378809	0.413545	5.75224	8.81E-09	0.00
1706	*C20orf56*	442.325	2.378758	0.415571	5.724066	1.04E-08	0.00
6312	*GOLGA6L6*	2.145727	2.378258	0.734146	3.23949	0.001197	0.00
1657	*TMED6*	109.0493	2.377577	0.412061	5.769956	7.93E-09	0.00
1180	*DMRTC1B*	25.93194	2.372747	0.375563	6.31784	2.65E-10	0.00
750	*GREB1*	286.9331	2.364281	0.335172	7.053941	1.74E-12	0.00
3120	*HS3ST5*	16.19361	2.363763	0.50619	4.669717	3.02E-06	0.00
305	*TMPRSS2*	2722.938	2.35907	0.282411	8.353317	6.64E-17	0.00
2950	*CLDN8*	67.49457	2.355467	0.493513	4.772854	1.82E-06	0.00
119	*GPR116*	5087.836	2.353062	0.24192	9.726605	2.32E-22	0.00
371	*SCNN1B*	1289.182	2.349601	0.289111	8.126978	4.40E-16	0.00
2919	*CYP4Z2P*	4.132789	2.345979	0.490377	4.784037	1.72E-06	0.00
2154	*GLRA3*	6.155108	2.34419	0.442243	5.300681	1.15E-07	0.00
289	*PNPLA7*	208.8334	2.342539	0.277733	8.434508	3.33E-17	0.00
1405	*SFTPB*	194783.2	2.341615	0.387148	6.048369	1.46E-09	0.00
1797	*CRYM*	542.1285	2.334395	0.415371	5.620019	1.91E-08	0.00
3217	*MAP3K15*	16.65281	2.332872	0.50529	4.616899	3.90E-06	0.00
801	*BAI1*	72.92377	2.329259	0.335277	6.947272	3.72E-12	0.00
4239	*OXT*	1.798462	2.325707	0.565387	4.113475	3.90E-05	0.00
2861	*ZSCAN4*	8.172285	2.322808	0.481768	4.821429	1.43E-06	0.00
2065	*ORM1*	497.4354	2.319834	0.43236	5.365514	8.07E-08	0.00
1011	*LOC283174*	166.9659	2.314613	0.352924	6.558384	5.44E-11	0.00
499	*GPR133*	763.8975	2.306407	0.299535	7.699967	1.36E-14	0.00
1518	*MST1P9*	537.7061	2.306126	0.390613	5.903859	3.55E-09	0.00
3073	*MUC21*	2290.274	2.304543	0.490105	4.702142	2.57E-06	0.00
6812	*SPP2*	8.03013	2.303866	0.756722	3.044533	0.00233	0.01
1806	*ZNF385B*	352.4725	2.296147	0.409207	5.61121	2.01E-08	0.00
2595	*ZACN*	1.584548	2.294518	0.460567	4.981941	6.29E-07	0.00
1835	*GGTLC1*	304.5147	2.292947	0.410605	5.584312	2.35E-08	0.00
1514	*KHDRBS2*	19.3201	2.292635	0.388133	5.906826	3.49E-09	0.00
10707	*OPRD1*	0.666558	2.290333	1.228896	1.863733	0.062359	0.11
9531	*SST*	3.620468	2.288884	1.047471	2.185152	0.028878	0.06
130	*DAAM2*	596.0459	2.281373	0.238483	9.566195	1.11E-21	0.00
3445	*SHISA3*	360.0324	2.279652	0.507781	4.489443	7.14E-06	0.00
1490	*ERBB4*	71.68318	2.26961	0.382438	5.934577	2.95E-09	0.00
2251	*ELF5*	283.888	2.269486	0.434553	5.222578	1.76E-07	0.00
5622	*FOXN4*	4.830339	2.266173	0.643331	3.522561	0.000427	0.00
459	*SLC47A1*	235.4363	2.261138	0.289253	7.817156	5.40E-15	0.00
5144	*OPRK1*	28.62725	2.257569	0.607691	3.714993	0.000203	0.00
476	*NRXN3*	154.1759	2.255139	0.289866	7.779929	7.26E-15	0.00
1169	*LRRC36*	45.89014	2.253381	0.355607	6.336717	2.35E-10	0.00
3586	*ORM2*	300.6292	2.252129	0.509495	4.420315	9.86E-06	0.00
1594	*KCTD19*	7.895438	2.252086	0.386544	5.826214	5.67E-09	0.00
5666	*GPR12*	2.050014	2.251615	0.642841	3.502601	0.000461	0.00
201	*EPHX1*	10069.04	2.249549	0.249962	8.999576	2.27E-19	0.00
1943	*CALML6*	3.211695	2.244216	0.410815	5.462832	4.69E-08	0.00
5809	*CHGA*	13.60574	2.241689	0.651354	3.441583	0.000578	0.00
318	*EFR3B*	206.9641	2.241424	0.270039	8.300373	1.04E-16	0.00
5407	*KLK12*	23.31931	2.238752	0.620876	3.605796	0.000311	0.00
2360	*LPPR1*	121.4679	2.230727	0.43395	5.140517	2.74E-07	0.00
1249	*FAM183B*	4.372857	2.228869	0.357554	6.233659	4.56E-10	0.00
2009	*SCN4A*	23.31247	2.224037	0.411105	5.409904	6.31E-08	0.00
177	*IRX3*	1109.518	2.223969	0.24103	9.226946	2.78E-20	0.00
3591	*AQP5*	1187.75	2.222646	0.50314	4.417552	9.98E-06	0.00
5179	*GPHA2*	2.082851	2.215732	0.59959	3.69541	0.00022	0.00
1105	*HSPB9*	9.297331	2.214466	0.344614	6.425935	1.31E-10	0.00
2766	*TMC2*	3.877853	2.210545	0.45351	4.874307	1.09E-06	0.00

2338	*PPP1R1B*	1165.871	2.206145	0.427833	5.156561	2.52E-07	0.00
745	*CDKL2*	362.2486	2.203346	0.311912	7.064004	1.62E-12	0.00
816	*COLEC11*	44.03056	2.202097	0.317821	6.928738	4.25E-12	0.00
1046	*C11orf92*	300.9837	2.201107	0.33814	6.509447	7.54E-11	0.00
4089	*HGFAC*	7.43451	2.194903	0.525285	4.178502	2.93E-05	0.00
10231	*AGXT2*	0.828656	2.194424	1.104392	1.986998	0.046923	0.08
1252	*FREM2*	455.0012	2.192681	0.351999	6.229228	4.69E-10	0.00
690	*DLX3*	93.39034	2.187381	0.305272	7.165356	7.76E-13	0.00
4550	*CLDN2*	1379.817	2.185752	0.550477	3.970652	7.17E-05	0.00
1269	*ALOX15B*	1588.829	2.184307	0.351539	6.213561	5.18E-10	0.00
4748	*TMEM72*	0.927475	2.183079	0.561469	3.888157	0.000101	0.00
1927	*PEG10*	3336.005	2.181891	0.397761	5.485433	4.12E-08	0.00
7816	*OTC*	0.888062	2.18091	0.80217	2.718764	0.006553	0.02
5848	*T*	5.137106	2.180593	0.636745	3.424596	0.000616	0.00
3467	*PAK3*	32.16931	2.173166	0.484937	4.481334	7.42E-06	0.00
380	*RAP1GAP*	2032.419	2.170737	0.268565	8.082724	6.33E-16	0.00
3458	*CNTN2*	2.466511	2.168663	0.483541	4.484962	7.29E-06	0.00
2677	*CYP4B1*	2457.283	2.165166	0.438705	4.93536	8.00E-07	0.00
5936	*MYH1*	0.88049	2.164096	0.638237	3.390741	0.000697	0.00
394	*TFAP2E*	56.84792	2.158746	0.268466	8.04103	8.91E-16	0.00
1245	*CEL*	31.83205	2.138693	0.342945	6.23626	4.48E-10	0.00
1122	*CAC1F*	24.80164	2.138411	0.333658	6.408997	1.46E-10	0.00
1008	*ACOXL*	58.73982	2.13829	0.325923	6.560729	5.35E-11	0.00
365	*C1orf116*	5427.211	2.137769	0.262551	8.142294	3.88E-16	0.00
826	*PALM3*	225.4196	2.136916	0.309231	6.910424	4.83E-12	0.00
9252	*COL20A1*	0.604044	2.134316	0.941506	2.266918	0.023395	0.05
7277	*HAND1*	2.253817	2.131942	0.737065	2.892477	0.003822	0.01
2203	*LOC284578*	59.46281	2.129871	0.404953	5.259556	1.44E-07	0.00
1016	*NKX2-1*	2953.96	2.128732	0.325149	6.546945	5.87E-11	0.00
3660	*C1orf230*	9.772133	2.12829	0.485878	4.380294	1.19E-05	0.00
1536	*B3GAT1*	69.35759	2.125805	0.361269	5.884265	4.00E-09	0.00
2180	*CAPN9*	152.4005	2.124688	0.402711	5.275957	1.32E-07	0.00
432	*PYGM*	21.55105	2.123867	0.268499	7.91015	2.57E-15	0.00
1345	*HOXD1*	260.417	2.122957	0.346625	6.12465	9.09E-10	0.00
3143	*RXRG*	19.90449	2.122301	0.455633	4.65792	3.19E-06	0.00
5032	*LOC149620*	12.90808	2.120565	0.563681	3.761993	0.000169	0.00
2199	*HES5*	3.774698	2.115102	0.401874	5.263099	1.42E-07	0.00
671	*CBR1*	6298.944	2.114887	0.293775	7.19899	6.07E-13	0.00
424	*ESYT3*	235.0574	2.111342	0.266009	7.93712	2.07E-15	0.00
3446	*GDF7*	1.184226	2.110184	0.470074	4.489047	7.15E-06	0.00
7567	*MRGPRE*	2.383291	2.109808	0.755461	2.792743	0.005226	0.01
6238	*LOC284798*	6.076368	2.109394	0.645529	3.267701	0.001084	0.00
757	*CAC1D*	204.7452	2.105791	0.298878	7.045648	1.85E-12	0.00
820	*SUSD4*	342.684	2.097871	0.302866	6.926722	4.31E-12	0.00
329	*KCNJ11*	137.1749	2.092046	0.253562	8.250635	1.58E-16	0.00
495	*SYP*	70.13942	2.089548	0.271033	7.709576	1.26E-14	0.00
3261	*FAM184B*	8.168743	2.089149	0.45537	4.587808	4.48E-06	0.00
571	*ICAM5*	175.9877	2.086606	0.280105	7.449364	9.38E-14	0.00
1069	*MST1P2*	353.446	2.083536	0.322163	6.467325	9.98E-11	0.00
1413	*IRX2*	630.7027	2.081525	0.344551	6.041277	1.53E-09	0.00
2547	*RNF222*	3.295655	2.080502	0.414588	5.018235	5.21E-07	0.00
12963	*CLEC2A*	0.535636	2.080451	1.65015	1.260765	0.207393	0.29
1955	*SOX2*	314.2331	2.078458	0.381394	5.449637	5.05E-08	0.00
2628	*FER1L5*	7.861492	2.077445	0.418247	4.967029	6.80E-07	0.00
1004	*C1orf127*	21.49034	2.077325	0.316136	6.570982	5.00E-11	0.00
2612	*SMCR5*	1.018199	2.072837	0.416778	4.973479	6.58E-07	0.00
2021	*HPCAL4*	34.95275	2.072535	0.38346	5.404825	6.49E-08	0.00
695	*SLC16A11*	50.64247	2.07144	0.289623	7.152186	8.54E-13	0.00
7076	*RFX4*	0.867433	2.067915	0.700596	2.95165	0.003161	0.01
10143	*OTOR*	0.481332	2.064266	1.026451	2.01107	0.044318	0.08
5068	*DDC*	183.2512	2.062736	0.550231	3.748856	0.000178	0.00
421	*TPPP*	438.8029	2.061985	0.259044	7.959991	1.72E-15	0.00
18921	*INSL6*	0.389998	2.061489	2.605551	0.791191	0.428832	NA

6230	*CHRM2*	0.682294	2.05486	0.628282	3.270601	0.001073	0.00
782	*CECR2*	133.8416	2.051779	0.293902	6.981161	2.93E-12	0.00
3801	*PITX2*	144.5296	2.04556	0.474261	4.313152	1.61E-05	0.00
152	*FAM69B*	464.851	2.043216	0.217139	9.4097	4.98E-21	0.00
1548	*ALS2CR11*	35.1051	2.039641	0.347635	5.867192	4.43E-09	0.00
2749	*C9orf106*	1.562961	2.038232	0.417461	4.882451	1.05E-06	0.00
513	*FAM189A2*	179.6764	2.034366	0.265701	7.656594	1.91E-14	0.00
3597	*SERPINC1*	3.069715	2.034237	0.460676	4.415768	1.01E-05	0.00
9256	*LOC440040*	0.642107	2.029822	0.895704	2.266174	0.023441	0.05
8646	*PHOX2B*	1.545148	2.026904	0.826388	2.452727	0.014178	0.03
4148	*EM*	32.04779	2.024368	0.48765	4.151275	3.31E-05	0.00
5311	*KRT40*	4.574911	2.022276	0.554859	3.644665	0.000268	0.00
10204	*OBP2B*	0.755028	2.021117	1.013711	1.99378	0.046176	0.08
1394	*TEKT5*	7.748454	2.02062	0.333371	6.061182	1.35E-09	0.00
983	*CLU*	12262.88	2.0167	0.305548	6.600275	4.10E-11	0.00
2282	*FCAMR*	6.166247	2.006435	0.385577	5.203717	1.95E-07	0.00
2837	*NTN3*	1.742829	2.003259	0.414242	4.835964	1.33E-06	0.00

Table S5. Upregulated (614 genes) expressed at > 2-fold in low risk group.

ENE ID	*Gene*	baseMean	log2FoldChange	lfcSE	stat	pvalue	padj
268	*PSG3*	11.30431	-8.43495	0.991614	-8.50629	1.80E-17	1.22E-15
39	*UPK1B*	588.4984	-8.20873	0.713108	-11.5112	1.16E-30	5.43E-28
754	*MAGEA4*	347.5992	-8.15665	1.156542	-7.05262	1.76E-12	4.26E-11
27	*NTS*	1532.419	-8.04162	0.669854	-12.005	3.34E-33	2.26E-30
144	*S100A7*	73.90454	-7.64487	0.807318	-9.46948	2.81E-21	3.57E-19
10	*NTSR1*	107.2943	-7.13438	0.550524	-12.9593	2.08E-38	3.81E-35
189	*EPS8L3*	127.6863	-7.01949	0.769581	-9.12119	7.43E-20	7.19E-18
168	*MUC2*	77.20751	-6.97876	0.74793	-9.33078	1.05E-20	1.14E-18
7	*KRT6A*	5350.933	-6.81282	0.514861	-13.2323	5.71E-40	1.49E-36
691	*TFF2*	43.58643	-6.80144	0.950233	-7.15766	8.21E-13	2.17E-11
5	*KRT6B*	636.3994	-6.72349	0.4972	-13.5227	1.15E-41	4.20E-38
13	*KRT6C*	317.5458	-6.71255	0.522628	-12.8438	9.31E-38	1.31E-34
104	*ANXA10*	117.7618	-6.42713	0.647607	-9.92443	3.26E-23	5.73E-21
17	*SERPINB7*	67.32402	-6.35058	0.500803	-12.6808	7.56E-37	8.12E-34
1289	*MAGEB2*	33.58366	-6.24035	1.008226	-6.18943	6.04E-10	8.56E-09
115	*DSG3*	91.40255	-6.09432	0.623076	-9.78101	1.36E-22	2.16E-20
959	*IRX4*	14.33123	-6.08028	0.914687	-6.64739	2.98E-11	5.68E-10
32	*RHCG*	74.66173	-6.02485	0.511842	-11.7709	5.51E-32	3.15E-29
468	*GABRA2*	13.35186	-5.9126	0.758031	-7.79994	6.19E-15	2.42E-13
229	*APOBEC1*	9.92733	-5.9002	0.673074	-8.76605	1.85E-18	1.48E-16
77	*CDHR2*	41.96052	-5.84165	0.571819	-10.2159	1.68E-24	3.99E-22
2907	*HOXD13*	12.08023	-5.82486	1.214794	-4.79494	1.63E-06	1.02E-05
69	*CIDEC*	15.8052	-5.80887	0.553816	-10.4888	9.73E-26	2.58E-23
2330	*CASP14*	7.873216	-5.80311	1.124201	-5.16198	2.44E-07	1.92E-06
482	*HOXA13*	7.399258	-5.77437	0.744465	-7.75639	8.74E-15	3.31E-13
58	*GDA*	148.2053	-5.64226	0.5246	-10.7554	5.59E-27	1.76E-24
173	*FGF5*	14.08947	-5.63808	0.608404	-9.26701	1.91E-20	2.02E-18
7097	*CT45A3*	5.908607	-5.56884	1.889816	-2.94676	0.003211	0.00827
1074	*TM4SF20*	13.11907	-5.56766	0.861436	-6.46323	1.02E-10	1.74E-09
48	*BNC1*	43.93073	-5.49496	0.495068	-11.0994	1.26E-28	4.81E-26
12	*SERPINB5*	625.3392	-5.49333	0.426429	-12.8822	5.67E-38	8.64E-35
65	*PSCA*	906.3812	-5.48944	0.515634	-10.646	1.82E-26	5.12E-24
3	*TRIM29*	1659.321	-5.48601	0.365973	-14.9902	8.51E-51	5.18E-47
601	*UNC5D*	28.4424	-5.42476	0.736627	-7.36433	1.78E-13	5.41E-12
863	*C12orf39*	42.91619	-5.40733	0.793098	-6.81798	9.23E-12	1.96E-10
1084	*FOXL2*	8.687227	-5.40355	0.837674	-6.45066	1.11E-10	1.88E-09
567	*SPRR3*	46.3677	-5.37763	0.720796	-7.46069	8.61E-14	2.78E-12
212	*PAPL*	9.500783	-5.23245	0.585888	-8.9308	4.23E-19	3.65E-17
2980	*PSG4*	4.186403	-5.1915	1.090858	-4.7591	1.94E-06	1.19E-05
355	*TFF1*	983.0174	-5.15743	0.631326	-8.16919	3.10E-16	1.60E-14
494	*SLC6A15*	40.06093	-5.08234	0.658782	-7.71476	1.21E-14	4.48E-13
3862	*CT45A5*	4.51691	-4.99362	1.165672	-4.2839	1.84E-05	8.69E-05

200	*IGFBP1*	63.82404	-4.93433	0.547867	-9.00643	2.13E-19	1.95E-17
237	*KRT14*	167.0053	-4.8979	0.561547	-8.72215	2.73E-18	2.11E-16
4463	*LIN28B*	12.1964	-4.8865	1.218617	-4.00987	6.08E-05	0.000249
3541	*CT45A1*	28.136	-4.87073	1.095884	-4.44456	8.81E-06	4.54E-05
28	*ANXA8*	561.1963	-4.85926	0.404967	-11.9991	3.59E-33	2.34E-30
544	*SPRR2D*	68.03542	-4.85126	0.642466	-7.551	4.32E-14	1.45E-12
402	*TCN1*	712.8942	-4.84528	0.604015	-8.02178	1.04E-15	4.74E-14
496	*HOXA11*	18.55152	-4.77397	0.619277	-7.70894	1.27E-14	4.67E-13
8	*TNS4*	1473.005	-4.75853	0.362607	-13.1231	2.43E-39	5.55E-36
30	*RSPO3*	581.0651	-4.74807	0.400541	-11.8541	2.05E-32	1.25E-29
2057	*GPR128*	3.500437	-4.74461	0.882442	-5.37668	7.59E-08	6.74E-07
125	*SLC13A5*	36.43625	-4.6761	0.483487	-9.6716	3.98E-22	5.82E-20
3240	*SPRR2E*	3.998718	-4.62519	1.004034	-4.6066	4.09E-06	2.31E-05
9873	*GAGE12D*	17.98323	-4.61299	2.208103	-2.08912	0.036697	0.067934
214	*KRT16*	780.0609	-4.61216	0.516835	-8.92386	4.50E-19	3.85E-17
62	*MT1A*	66.87833	-4.58915	0.429748	-10.6787	1.28E-26	3.77E-24
133	*KRT81*	864.1101	-4.54546	0.477508	-9.51914	1.75E-21	2.40E-19
860	*SPRR1B*	228.6776	-4.53754	0.664739	-6.82605	8.73E-12	1.85E-10
26	*IL1A*	64.55838	-4.51634	0.375541	-12.0262	2.59E-33	1.82E-30
203	*BEST3*	4.386154	-4.51338	0.501891	-8.99274	2.41E-19	2.17E-17
71	*TRPA1*	32.03626	-4.51117	0.433274	-10.4118	2.19E-25	5.64E-23
4	*GJB3*	506.5518	-4.49431	0.322611	-13.9311	4.10E-44	1.87E-40
72	*PRSS3*	98.8677	-4.47112	0.431396	-10.3643	3.60E-25	9.15E-23
1447	*TRIM40*	5.194868	-4.45747	0.742858	-6.00044	1.97E-09	2.49E-08
154	*ARL14*	54.93069	-4.45263	0.47393	-9.39511	5.72E-21	6.70E-19
160	*KRT5*	563.9812	-4.45033	0.474811	-9.37284	7.06E-21	8.07E-19
771	*C12orf36*	56.33558	-4.44839	0.634601	-7.00974	2.39E-12	5.66E-11
5565	*COX7B2*	12.69306	-4.44676	1.255105	-3.54294	0.000396	0.0013
192	*LOC554202*	58.5482	-4.44601	0.489369	-9.08519	1.04E-19	9.85E-18
117	*GJB4*	34.3854	-4.44284	0.455484	-9.75412	1.77E-22	2.76E-20
1882	*FAM25A*	2.355634	-4.41711	0.798169	-5.53405	3.13E-08	3.04E-07
8760	*GAGE12J*	2.921452	-4.41445	1.823333	-2.42109	0.015474	0.032285
29	*CXCL5*	776.6156	-4.41368	0.371069	-11.8945	1.26E-32	7.97E-30
171	*UCA1*	132.822	-4.41163	0.475564	-9.27664	1.75E-20	1.87E-18
215	*GAL*	80.882	-4.41158	0.495114	-8.91024	5.09E-19	4.33E-17
358	*SERPINB4*	70.44919	-4.35924	0.533954	-8.16407	3.24E-16	1.65E-14
1032	*LIPK*	3.946511	-4.35885	0.667185	-6.53319	6.44E-11	1.14E-09
8525	*DPPA2*	3.321874	-4.34666	1.745886	-2.48966	0.012786	0.027413
823	*TMPRSS11D*	9.42722	-4.33204	0.626144	-6.91861	4.56E-12	1.01E-10
3798	*CGA*	38.98661	-4.3266	1.002684	-4.31502	1.60E-05	7.68E-05
396	*ANXA13*	13.8205	-4.30236	0.535457	-8.03493	9.36E-16	4.32E-14
6	*VNN1*	293.2833	-4.27696	0.322158	-13.276	3.19E-40	9.72E-37
543	*SERPINB3*	255.1123	-4.26398	0.564267	-7.55668	4.13E-14	1.39E-12
5157	*MAGEA9B*	112.6833	-4.25621	1.14763	-3.70869	0.000208	0.000738
8047	*KRT31*	2.711597	-4.24072	1.602562	-2.64621	0.00814	0.018488
63	*KLRC3*	23.22492	-4.24024	0.397326	-10.6719	1.38E-26	4.00E-24
2434	*HOXD11*	12.29222	-4.20683	0.826077	-5.09255	3.53E-07	2.65E-06
196	*C14orf34*	5.504869	-4.18614	0.46251	-9.05091	1.42E-19	1.32E-17
57	*ANXA8L2*	312.3685	-4.1764	0.388175	-10.7591	5.37E-27	1.72E-24
1241	*NPFFR2*	13.97531	-4.16691	0.667598	-6.24165	4.33E-10	6.38E-09
12326	*TSPY3*	1.802924	-4.16173	2.914263	-1.42806	0.153276	0.227277
4111	*SPANXC*	1.942776	-4.1385	0.99273	-4.16881	3.06E-05	0.000136
315	*LOC100216001*	11.38965	-4.13018	0.497237	-8.30626	9.88E-17	5.71E-15
8287	*PASD1*	1.92952	-4.12811	1.605523	-2.5712	0.010135	0.022352
4293	*MAGEC2*	83.42906	-4.1201	1.008846	-4.08397	4.43E-05	0.000188
1733	*SPRR2A*	16.14383	-4.10182	0.720798	-5.69066	1.27E-08	1.33E-07
1130	*MAGEA8*	9.47471	-4.09817	0.640662	-6.39678	1.59E-10	2.57E-09
1297	*PADI3*	26.7311	-4.08945	0.66163	-6.18087	6.37E-10	8.98E-09
1368	*KNG1*	17.28045	-4.08281	0.670521	-6.08901	1.14E-09	1.52E-08
181	*TRIM31*	327.8162	-4.077	0.443597	-9.19079	3.90E-20	3.94E-18
3989	*SAGE1*	3.41053	-4.0747	0.963837	-4.22758	2.36E-05	0.000108
1168	*KLK6*	309.1095	-4.07431	0.642911	-6.33729	2.34E-10	3.66E-09
183	*PI3*	336.8848	-4.06248	0.443021	-9.16996	4.73E-20	4.73E-18

3418	*GAST*	1.841672	-4.0597	0.901948	-4.50104	6.76E-06	3.62E-05
9212	*TGIF2LX*	1.649058	-4.03321	1.768279	-2.28087	0.022556	0.044753
202	*GUCY2C*	24.22022	-4.03081	0.447998	-8.99738	2.31E-19	2.09E-17
161	*WNT7A*	55.25395	-4.01242	0.428747	-9.35848	8.09E-21	9.18E-19
15	*S100A8*	1039.77	-4.00681	0.312843	-12.8077	1.48E-37	1.81E-34
9968	*GAGE8*	1.660216	-3.97576	1.928767	-2.0613	0.039275	0.072013
1837	*MUCL1*	26.40695	-3.96047	0.709424	-5.58265	2.37E-08	2.36E-07
3028	*DSG4*	1.718254	-3.95817	0.836693	-4.73073	2.24E-06	1.35E-05
136	*CALB2*	118.8928	-3.95813	0.416012	-9.51446	1.83E-21	2.44E-19
1464	*KLK5*	36.61488	-3.94751	0.660334	-5.97805	2.26E-09	2.82E-08
22	*AKAP12*	3141.173	-3.94695	0.323585	-12.1976	3.20E-34	2.66E-31
5057	*LOC100133469*	3.842232	-3.94248	1.050713	-3.7522	0.000175	0.000634
485	*OLFM4*	28.19618	-3.94059	0.508828	-7.74444	9.60E-15	3.62E-13
4516	*VAX1*	8.208541	-3.9365	0.988185	-3.98357	6.79E-05	0.000275
6077	*UGT1A7*	1.832363	-3.91972	1.175519	-3.33446	0.000855	0.002571
872	*TRIM15*	43.80266	-3.91829	0.57612	-6.80118	1.04E-11	2.17E-10
827	*RAET1L*	2.158147	-3.90143	0.56475	-6.90824	4.91E-12	1.08E-10
55	*KLRC2*	37.45724	-3.89943	0.361402	-10.7897	3.85E-27	1.28E-24
5002	*PSG1*	2.237944	-3.88698	1.029461	-3.77574	0.00016	0.000583
4767	*ARHGAP36*	2.046348	-3.88603	1.001832	-3.87893	0.000105	0.000402
5509	*SPANXE*	1.932374	-3.86652	1.084671	-3.56469	0.000364	0.001209
73	*SH2D5*	25.87345	-3.84979	0.372083	-10.3466	4.34E-25	1.09E-22
367	*DKK1*	877.4378	-3.82537	0.470126	-8.1369	4.06E-16	2.02E-14
1136	*A2ML1*	19.58881	-3.82452	0.59872	-6.38783	1.68E-10	2.71E-09
808	*INHA*	498.6808	-3.81052	0.549361	-6.93628	4.03E-12	9.11E-11
3841	*RTL1*	1.776059	-3.80898	0.886963	-4.29441	1.75E-05	8.33E-05
4499	*MRGPRX3*	1.548847	-3.80505	0.953115	-3.99223	6.55E-05	0.000266
502	*C11orf86*	88.35623	-3.79644	0.493317	-7.69574	1.41E-14	5.12E-13
4774	*GUCA2B*	2.958707	-3.79591	0.979455	-3.87553	0.000106	0.000407
3330	*CRCT1*	2.479514	-3.78577	0.8312	-4.55458	5.25E-06	2.88E-05
280	*MYH16*	8.123721	-3.77447	0.446274	-8.45774	2.73E-17	1.78E-15
3140	*CGB8*	4.33702	-3.76301	0.807768	-4.65853	3.18E-06	1.85E-05
5829	*MGC34034*	1.562945	-3.7525	1.092849	-3.43369	0.000595	0.001867
4287	*LHX1*	5.031889	-3.7515	0.918155	-4.08591	4.39E-05	0.000187
1	*ARNTL2*	429.0032	-3.74855	0.233513	-16.0528	5.46E-58	9.98E-54
1303	*TRY6*	23.378	-3.7447	0.606629	-6.17296	6.70E-10	9.40E-09
7288	*DCAF4L2*	2.741596	-3.7444	1.296991	-2.88699	0.003889	0.009754
98	*IL20RB*	311.1925	-3.73894	0.374813	-9.9755	1.95E-23	3.64E-21
343	*GABRP*	118.7837	-3.73601	0.454913	-8.21257	2.16E-16	1.15E-14
2993	*GIP*	1.843178	-3.73123	0.78489	-4.75382	2.00E-06	1.22E-05
53	*C12orf70*	6.144973	-3.72766	0.34404	-10.835	2.35E-27	8.11E-25
1928	*LGALS7B*	28.66681	-3.72495	0.679279	-5.48368	4.17E-08	3.95E-07
2991	*GPR78*	2.704021	-3.72439	0.7832	-4.75535	1.98E-06	1.21E-05
397	*DSC3*	107.4304	-3.70823	0.461537	-8.03452	9.39E-16	4.32E-14
174	*UCN2*	19.22386	-3.70072	0.399465	-9.26421	1.97E-20	2.06E-18
904	*SYT13*	310.8231	-3.67713	0.54597	-6.73504	1.64E-11	3.31E-10
3233	*NR1H4*	21.12607	-3.67481	0.796995	-4.61084	4.01E-06	2.27E-05
2077	*ACTL8*	5.448364	-3.63494	0.678378	-5.35829	8.40E-08	7.39E-07
792	*FER1L6*	8.32946	-3.63233	0.521432	-6.96607	3.26E-12	7.52E-11
6880	*PSG9*	1.793352	-3.62452	1.200793	-3.01844	0.002541	0.00675
292	*SAA2*	269.3483	-3.61229	0.430535	-8.39023	4.85E-17	3.04E-15
3356	*PAQR9*	2.318743	-3.61189	0.79616	-4.53664	5.72E-06	3.11E-05
24	*RAET1E*	40.80162	-3.60809	0.299474	-12.0481	1.98E-33	1.51E-30
1949	*CLC*	11.70815	-3.60215	0.659846	-5.45907	4.79E-08	4.49E-07
8434	*A1CF*	1.252661	-3.56759	1.414629	-2.52193	0.011671	0.025293
127	*KCNF1*	43.95226	-3.55996	0.368595	-9.65819	4.54E-22	6.53E-20
892	*HMGA2*	604.8333	-3.55309	0.525296	-6.76398	1.34E-11	2.75E-10
2274	*SPRR2F*	5.552491	-3.547	0.681083	-5.20788	1.91E-07	1.54E-06
97	*MT1M*	122.4631	-3.54435	0.354586	-9.99574	1.59E-23	3.00E-21
565	*PTPRN*	85.85762	-3.54426	0.474778	-7.4651	8.32E-14	2.69E-12
255	*KRT17*	5138.368	-3.54406	0.41223	-8.59731	8.16E-18	5.85E-16
251	*DNER*	364.8138	-3.53541	0.408797	-8.64834	5.23E-18	3.81E-16
3269	*OR1F1*	1.769146	-3.53489	0.770905	-4.58538	4.53E-06	2.53E-05

631	*EREG*	608.0579	-3.53371	0.484249	-7.29728	2.94E-13	8.51E-12
2393	*NBPF4*	2.483645	-3.52938	0.68958	-5.11815	3.09E-07	2.36E-06
994	*UGT2B7*	22.71686	-3.50467	0.532328	-6.58368	4.59E-11	8.44E-10
1217	*PRSS1*	36.67972	-3.48921	0.55553	-6.28085	3.37E-10	5.06E-09
5682	*S100A7A*	1.719433	-3.48663	0.997681	-3.49473	0.000475	0.001526
911	*RPSAP52*	23.53397	-3.47656	0.51705	-6.72384	1.77E-11	3.55E-10
155	*GRAMD1B*	237.3862	-3.47466	0.369807	-9.39585	5.68E-21	6.70E-19
1340	*IFNE*	8.5912	-3.46622	0.565675	-6.12758	8.92E-10	1.22E-08
11791	*GAGE1*	6.075965	-3.46155	2.190849	-1.58	0.114106	0.176874
45	*RARRES1*	1316.381	-3.45966	0.307895	-11.2365	2.70E-29	1.10E-26
1429	*GBX2*	4.39623	-3.45289	0.574108	-6.01435	1.81E-09	2.31E-08
1850	*C20orf141*	1.665407	-3.44363	0.618536	-5.56739	2.59E-08	2.55E-07
187	*CREG2*	9.055804	-3.44255	0.376777	-9.13683	6.43E-20	6.29E-18
3978	*UGT1A10*	9.00823	-3.4332	0.810869	-4.23397	2.30E-05	0.000105
404	*SAA1*	504.2271	-3.41129	0.42596	-8.00848	1.16E-15	5.25E-14
363	*PNPLA1*	6.725219	-3.40803	0.418453	-8.14436	3.81E-16	1.92E-14
1292	*TMEM195*	8.380312	-3.40552	0.550466	-6.18661	6.15E-10	8.70E-09
517	*SERPINB2*	22.88408	-3.40255	0.445509	-7.63744	2.22E-14	7.83E-13
4021	*CDX2*	39.02575	-3.40122	0.807515	-4.21196	2.53E-05	0.000115
5049	*PRDM9*	2.011024	-3.39636	0.90428	-3.75587	0.000173	0.000625
738	*MYO3A*	19.7489	-3.39223	0.479439	-7.0754	1.49E-12	3.69E-11
1925	*HOXA11AS*	4.697486	-3.38276	0.616418	-5.48777	4.07E-08	3.86E-07
34	*SH3TC2*	37.99116	-3.38262	0.287999	-11.7453	7.47E-32	4.01E-29
272	*HOXA9*	28.28745	-3.37634	0.397808	-8.48735	2.11E-17	1.42E-15
4948	*SPRR2C*	12.07205	-3.3758	0.888158	-3.8009	0.000144	0.000533
603	*CLCA2*	51.3101	-3.37381	0.458251	-7.36235	1.81E-13	5.48E-12
3821	*FOXG1*	4.342601	-3.36322	0.78119	-4.30525	1.67E-05	7.98E-05
1247	*ABCC2*	706.4595	-3.3611	0.539101	-6.23464	4.53E-10	6.64E-09
1959	*IGFL1*	8.236303	-3.35178	0.615266	-5.44769	5.10E-08	4.76E-07
4186	*SLC1A6*	3.394993	-3.33461	0.805993	-4.13727	3.51E-05	0.000153
5247	*KRT34*	1.51423	-3.29937	0.899372	-3.66853	0.000244	0.00085
5146	*F2*	25.28868	-3.28841	0.885635	-3.71305	0.000205	0.000727
1314	*GYS2*	5.943942	-3.28592	0.532764	-6.16768	6.93E-10	9.63E-09
5428	*FBXL21*	2.028595	-3.2855	0.913557	-3.59639	0.000323	0.001086
3336	*LOC731789*	8.809947	-3.28313	0.721568	-4.54999	5.36E-06	2.94E-05
11336	*CT45A4*	1.128313	-3.27424	1.931902	-1.69483	0.090108	0.145269
4208	*C3orf72*	3.785013	-3.26235	0.790486	-4.12702	3.67E-05	0.00016
4694	*KRT75*	4.205377	-3.25039	0.831147	-3.91072	9.20E-05	0.000358
7331	*TAC1*	1.403971	-3.24825	1.131816	-2.86995	0.004105	0.010235
436	*COL4A6*	79.57737	-3.24595	0.412184	-7.87501	3.41E-15	1.43E-13
447	*CHST4*	44.19297	-3.24515	0.413769	-7.84291	4.40E-15	1.80E-13
5704	*NKX2-5*	4.273725	-3.24265	0.930507	-3.48481	0.000492	0.001578
6809	*C10orf99*	1.659639	-3.24061	1.064232	-3.04502	0.002327	0.006245
608	*GJB5*	81.5341	-3.23843	0.440532	-7.3512	1.96E-13	5.91E-12
2875	*B4GALNT2*	93.40233	-3.23734	0.672711	-4.81237	1.49E-06	9.48E-06
4094	*ALX1*	9.952714	-3.23325	0.774114	-4.17671	2.96E-05	0.000132
841	*TGM4*	3.188772	-3.2279	0.469891	-6.86946	6.44E-12	1.40E-10
14	*LAMC2*	10126.67	-3.22511	0.25162	-12.8174	1.31E-37	1.71E-34
7692	*SPANXA2*	1.02872	-3.20948	1.163165	-2.75927	0.005793	0.013765
321	*MYO7B*	41.04378	-3.20351	0.386505	-8.28841	1.15E-16	6.53E-15
940	*CLCN1*	5.175472	-3.20116	0.47915	-6.68091	2.37E-11	4.62E-10
1485	*TRIM10*	9.235498	-3.18847	0.536818	-5.93958	2.86E-09	3.52E-08
1944	*TH*	6.365829	-3.17784	0.581906	-5.46109	4.73E-08	4.45E-07
2440	*FGA*	14635.7	-3.1754	0.623901	-5.08959	3.59E-07	2.69E-06
483	*MYO1A*	13.07271	-3.17487	0.409424	-7.75447	8.87E-15	3.36E-13
1502	*NEFL*	45.67143	-3.17277	0.535678	-5.9229	3.16E-09	3.85E-08
551	*SERPI5*	113.3526	-3.17227	0.422117	-7.51515	5.68E-14	1.89E-12
7646	*PSG6*	0.900466	-3.1598	1.139924	-2.77194	0.005572	0.01332
3190	*DEFB4A*	6.166335	-3.15729	0.681498	-4.63286	3.61E-06	2.07E-05
2	*CD109*	1099.129	-3.15494	0.209385	-15.0677	2.64E-51	2.41E-47
1294	*LOC440173*	28.61087	-3.15198	0.509627	-6.18488	6.22E-10	8.78E-09
60	*GPR97*	74.5802	-3.14647	0.293833	-10.7084	9.30E-27	2.83E-24
9612	*DKFZp686A1627*	1.41332	-3.12867	1.448303	-2.16023	0.030755	0.058473

7494	*C17orf73*	1.224473	-3.11117	1.104745	-2.81619	0.00486	0.011852
6207	*UGT1A8*	2.0617	-3.10792	0.948491	-3.2767	0.00105	0.003093
407	*AMDHD1*	62.55429	-3.09842	0.387427	-7.99744	1.27E-15	5.70E-14
217	*TNFRSF6B*	571.4497	-3.09612	0.348136	-8.89343	5.93E-19	4.99E-17
1611	*ISM2*	6.459086	-3.09608	0.532747	-5.81153	6.19E-09	7.02E-08
278	*S100A2*	1176.903	-3.09548	0.365876	-8.46045	2.66E-17	1.75E-15
700	*CPA4*	11.1296	-3.09441	0.433824	-7.13288	9.83E-13	2.57E-11
6762	*MAGEA11*	3.456936	-3.09041	1.007594	-3.06712	0.002161	0.005842
137	*CDA*	536.4518	-3.08965	0.324682	-9.51591	1.80E-21	2.44E-19
37	*FOSL1*	425.9303	-3.08612	0.266607	-11.5755	5.48E-31	2.71E-28
2938	*PRDM13*	5.313038	-3.07776	0.644331	-4.77668	1.78E-06	1.11E-05
678	*ANO3*	97.8706	-3.07644	0.428365	-7.18182	6.88E-13	1.85E-11
1064	*NDP*	15.97211	-3.07293	0.474501	-6.47613	9.41E-11	1.62E-09
49	*IL1R2*	123.7396	-3.06872	0.278816	-11.0063	3.56E-28	1.33E-25
19	*SLC16A1*	868.3354	-3.06292	0.245872	-12.4574	1.27E-35	1.23E-32
1888	*PDZD3*	7.394902	-3.06206	0.553818	-5.529	3.22E-08	3.12E-07
339	*LRRC66*	25.03783	-3.06065	0.372288	-8.22119	2.01E-16	1.08E-14
5456	*SLC30A10*	1.288785	-3.0522	0.850971	-3.58673	0.000335	0.001122
184	*NIPAL4*	17.24877	-3.05174	0.333528	-9.14989	5.70E-20	5.64E-18
1351	*POPDC3*	93.02052	-3.04974	0.49882	-6.11392	9.72E-10	1.32E-08
842	*CYP2C9*	16.90149	-3.04968	0.444129	-6.86665	6.57E-12	1.43E-10
176	*LYPD3*	887.0304	-3.04876	0.330342	-9.22909	2.73E-20	2.83E-18
6455	*RPTN*	1.603927	-3.04691	0.956668	-3.18492	0.001448	0.0041
422	*KIR2DL4*	19.87252	-3.03556	0.381855	-7.9495	1.87E-15	8.11E-14
6791	*PAX3*	1.388205	-3.0287	0.992198	-3.05251	0.002269	0.006108
3165	*OR4C6*	1.800188	-3.0265	0.650657	-4.65146	3.30E-06	1.90E-05
10499	*PAGE2*	8.479361	-3.02032	1.57615	-1.91627	0.055331	0.096322
11876	*PSG2*	0.816801	-3.0186	1.939548	-1.55634	0.119627	0.184104
614	*S100A9*	12774.34	-3.01515	0.411273	-7.33126	2.28E-13	6.79E-12
9821	*GAGE4*	4.101033	-3.01342	1.432456	-2.10367	0.035407	0.065893
2145	*HRK*	2.020376	-3.01113	0.567304	-5.3078	1.11E-07	9.45E-07
21	*MT2A*	4565.438	-3.00759	0.245368	-12.2575	1.53E-34	1.33E-31
7313	*LCE3E*	0.851966	-3.00634	1.044717	-2.87765	0.004006	0.010013
4185	*CNTP4*	9.557261	-3.00445	0.726129	-4.13762	3.51E-05	0.000153
2705	*SPRR1A*	9.79943	-3.00144	0.610653	-4.91513	8.87E-07	5.99E-06
891	*CHR7*	6.244826	-2.98087	0.440586	-6.7657	1.33E-11	2.72E-10
3694	*ADH4*	4.269818	-2.98035	0.682605	-4.36614	1.26E-05	6.26E-05
100	*TM4SF19*	72.68005	-2.97779	0.298964	-9.96036	2.27E-23	4.15E-21
1604	*OLAH*	9.745122	-2.97715	0.511764	-5.81744	5.98E-09	6.81E-08
1740	*SYT9*	6.960516	-2.9767	0.523826	-5.68261	1.33E-08	1.39E-07
4084	*TRIML2*	1.472431	-2.97595	0.711876	-4.18044	2.91E-05	0.00013
101	*RGS20*	49.49259	-2.97354	0.298726	-9.95405	2.42E-23	4.38E-21
244	*KCNJ12*	114.9312	-2.97126	0.34152	-8.70008	3.32E-18	2.48E-16
865	*FAT2*	121.2816	-2.96711	0.435343	-6.81558	9.39E-12	1.98E-10
3174	*USH1C*	119.0713	-2.9565	0.636502	-4.64492	3.40E-06	1.96E-05
8463	*PSG5*	0.781657	-2.95546	1.17674	-2.51157	0.01202	0.025964
2475	*LINGO2*	4.102496	-2.95387	0.583396	-5.06323	4.12E-07	3.04E-06
5808	*CALML5*	13.57469	-2.94436	0.855437	-3.44194	0.000578	0.001817
2403	*CYP2C19*	6.806119	-2.94196	0.575537	-5.11168	3.19E-07	2.43E-06
900	*FZD10*	171.2934	-2.94088	0.435796	-6.74831	1.50E-11	3.03E-10
3557	*SERPINB13*	4.257508	-2.93553	0.662077	-4.43382	9.26E-06	4.76E-05
572	*TRIM58*	30.54497	-2.93043	0.3934	-7.44898	9.41E-14	3.01E-12
616	*FAM83B*	81.71308	-2.92764	0.399857	-7.32171	2.45E-13	7.26E-12
804	*LOC100127888*	19.8393	-2.92592	0.421456	-6.94241	3.85E-12	8.76E-11
4291	*NCR00162*	7.906629	-2.91922	0.714673	-4.0847	4.41E-05	0.000188
575	*SLC6A17*	39.29663	-2.91323	0.391573	-7.43981	1.01E-13	3.21E-12
13893	*GAGE2B*	6.176616	-2.91254	2.848132	-1.02261	0.30649	0.403204
6973	*FAM9C*	1.532022	-2.91116	0.97452	-2.98728	0.002815	0.007377
1947	*CDH17*	290.1117	-2.90807	0.532654	-5.45958	4.77E-08	4.48E-07
4257	*HBE1*	3.918498	-2.89431	0.705726	-4.10118	4.11E-05	0.000176
661	*CD177*	90.79625	-2.89347	0.400429	-7.22593	4.98E-13	1.38E-11
185	*RAET1G*	33.40851	-2.89138	0.31601	-9.14965	5.71E-20	5.64E-18
141	*PKP2*	633.3865	-2.88837	0.304474	-9.48644	2.39E-21	3.10E-19

139	*PKIB*	504.2694	-2.87216	0.301963	-9.51163	1.88E-21	2.47E-19
1371	*MMP3*	75.18893	-2.8718	0.471941	-6.08509	1.16E-09	1.55E-08
477	*S100A12*	13.40278	-2.8697	0.368915	-7.77875	7.32E-15	2.81E-13
2139	*MYPN*	4.792392	-2.86804	0.539646	-5.31466	1.07E-07	9.13E-07
35	*BIRC3*	2326.794	-2.86768	0.24652	-11.6327	2.81E-31	1.47E-28
2121	*CYP4F3*	161.3704	-2.86686	0.537736	-5.33135	9.75E-08	8.40E-07
123	*SLCO4A1*	375.5137	-2.8642	0.296081	-9.67371	3.90E-22	5.76E-20
4845	*LASS3*	1.911985	-2.85487	0.742236	-3.84631	0.00012	0.000452
2583	*SLC39A5*	10.93699	-2.84419	0.569473	-4.99443	5.90E-07	4.18E-06
3986	*CRP*	3.058555	-2.83973	0.671399	-4.22957	2.34E-05	0.000107
248	*KLRC1*	30.15127	-2.82576	0.326354	-8.65856	4.78E-18	3.52E-16
1012	*PPBP*	39.48397	-2.82321	0.430669	-6.5554	5.55E-11	1.00E-09
539	*COL7A1*	649.8948	-2.81844	0.372435	-7.5676	3.80E-14	1.29E-12
831	*FST*	178.7284	-2.8151	0.407923	-6.90105	5.16E-12	1.14E-10
623	*MT1H*	16.86945	-2.80387	0.38339	-7.31336	2.61E-13	7.64E-12
5923	*GABRA5*	2.532484	-2.80341	0.825388	-3.39647	0.000683	0.002106
210	*NUDT11*	32.51534	-2.79036	0.312236	-8.93671	4.01E-19	3.49E-17
481	*GPR1*	31.79354	-2.7812	0.358431	-7.75936	8.54E-15	3.24E-13
1682	*SAA4*	17.47699	-2.76851	0.481527	-5.74944	8.95E-09	9.73E-08
2476	*LRP1B*	14.61832	-2.76544	0.546287	-5.06225	4.14E-07	3.06E-06
8466	*ALPI*	0.840541	-2.76223	1.100315	-2.5104	0.012059	0.026035
5895	*LCN1*	1.072213	-2.7616	0.810763	-3.40618	0.000659	0.002043
1293	*AICDA*	5.536778	-2.75596	0.445487	-6.1864	6.16E-10	8.70E-09
1420	*PF4*	7.799756	-2.75527	0.45739	-6.02391	1.70E-09	2.19E-08
4883	*TP53TG3B*	4.352466	-2.75438	0.71911	-3.83027	0.000128	0.000479
86	*ASAM*	233.2919	-2.75318	0.272571	-10.1008	5.48E-24	1.16E-21
408	*AIM2*	215.0712	-2.74578	0.343462	-7.99441	1.30E-15	5.83E-14
3077	*MYBPC1*	18.75526	-2.73612	0.582331	-4.69856	2.62E-06	1.56E-05
3910	*CRISP3*	13.296	-2.73411	0.641466	-4.26228	2.02E-05	9.46E-05
471	*RELN*	88.93091	-2.73146	0.350708	-7.7884	6.79E-15	2.63E-13
948	*GBP6*	82.84807	-2.7213	0.408086	-6.66843	2.59E-11	4.98E-10
2537	*CALHM3*	4.365927	-2.71056	0.539589	-5.02338	5.08E-07	3.66E-06
3560	*KCNV1*	15.04711	-2.70313	0.610009	-4.43129	9.37E-06	4.81E-05
262	*FLNC*	808.7111	-2.69776	0.315996	-8.53732	1.37E-17	9.58E-16
2649	*PADI1*	57.10239	-2.69322	0.543425	-4.95601	7.20E-07	4.96E-06
2680	*KLK7*	57.17867	-2.68915	0.545194	-4.93246	8.12E-07	5.54E-06
2616	*KRT83*	31.3154	-2.6866	0.540407	-4.97144	6.65E-07	4.64E-06
680	*KYNU*	1538.136	-2.68368	0.373739	-7.18062	6.94E-13	1.86E-11
412	*LILRA3*	27.28448	-2.67641	0.335006	-7.98913	1.36E-15	6.03E-14
1526	*KRT23*	43.11372	-2.67493	0.454013	-5.89174	3.82E-09	4.58E-08
1692	*MAPK4*	165.9495	-2.66675	0.464402	-5.74234	9.34E-09	1.01E-07
4520	*CLCA4*	2.8282	-2.6628	0.668613	-3.98257	6.82E-05	0.000276
2116	*KHDC1L*	5.286516	-2.66118	0.499018	-5.33283	9.67E-08	8.35E-07
652	*PTHLH*	190.971	-2.65969	0.367245	-7.24229	4.41E-13	1.24E-11
4642	*GLP2R*	1.050767	-2.65399	0.674506	-3.93472	8.33E-05	0.000328
456	*CASP5*	6.913512	-2.65258	0.339003	-7.82463	5.09E-15	2.04E-13
1175	*ZG16B*	122.6273	-2.64949	0.418787	-6.32658	2.51E-10	3.90E-09
2257	*OR2W3*	2.122074	-2.64868	0.507467	-5.2194	1.80E-07	1.45E-06
995	*PROK2*	5.993098	-2.64462	0.401792	-6.58206	4.64E-11	8.52E-10
56	*TGFBI*	8878.612	-2.64203	0.244988	-10.7843	4.08E-27	1.33E-24
78	*TRIM7*	66.3318	-2.63681	0.258631	-10.1952	2.08E-24	4.88E-22
415	*CD70*	30.30913	-2.63657	0.330864	-7.96875	1.60E-15	7.06E-14
195	*GJB2*	1144.788	-2.63022	0.290563	-9.05216	1.40E-19	1.31E-17
800	*L1CAM*	77.93208	-2.62861	0.378242	-6.94956	3.66E-12	8.37E-11
1167	*GPR87*	328.0613	-2.62397	0.413987	-6.33829	2.32E-10	3.64E-09
849	*IL1RL1*	105.2165	-2.61827	0.382277	-6.84914	7.43E-12	1.60E-10
25	*SLC2A1*	5417.374	-2.59863	0.215763	-12.0439	2.09E-33	1.53E-30
1747	*SBSN*	14.36171	-2.59311	0.457195	-5.67178	1.41E-08	1.48E-07
288	*HOXA1*	41.3441	-2.58612	0.306609	-8.43456	3.32E-17	2.10E-15
5572	*TIG*	40.9842	-2.57918	0.728341	-3.54117	0.000398	0.001307
1663	*NCCRP1*	303.0522	-2.57741	0.447274	-5.76248	8.29E-09	9.11E-08
731	*COX6B2*	21.68934	-2.56863	0.362226	-7.09123	1.33E-12	3.32E-11
6163	*MAGEB6*	0.944709	-2.56765	0.779479	-3.29406	0.000988	0.002929

5530	*C10orf90*	8.831379	-2.56436	0.721202	-3.55567	0.000377	0.001246
682	*GPR115*	154.3004	-2.56235	0.356935	-7.17877	7.03E-13	1.89E-11
384	*HTR7*	20.37259	-2.56185	0.317406	-8.07119	6.96E-16	3.31E-14
259	*KCP*	69.32671	-2.5567	0.299234	-8.54415	1.29E-17	9.14E-16
1264	*LOC283404*	7.199045	-2.54915	0.410056	-6.21659	5.08E-10	7.35E-09
9882	*HSD3B1*	0.84833	-2.54678	1.220444	-2.08676	0.03691	0.068258
3748	*MYO18B*	5.500286	-2.54261	0.586163	-4.33771	1.44E-05	7.02E-05
6031	*FLJ44054*	0.928396	-2.53976	0.758111	-3.35012	0.000808	0.002447
226	*IL6*	201.3596	-2.53913	0.287892	-8.81974	1.15E-18	9.28E-17
10807	*FLJ36000*	1.159567	-2.53676	1.380192	-1.83797	0.066066	0.111733
3241	*LOC148824*	1.557033	-2.53378	0.550192	-4.60526	4.12E-06	2.32E-05
250	*CLEC4E*	99.58018	-2.53361	0.29286	-8.65127	5.09E-18	3.72E-16
194	*XIRP1*	28.63408	-2.53196	0.279513	-9.05847	1.32E-19	1.25E-17
3144	*SLC15A1*	135.9022	-2.52761	0.542663	-4.65779	3.20E-06	1.86E-05
10440	*GHRHR*	0.68353	-2.52289	1.305257	-1.93287	0.053252	0.093227
277	*SH2D1B*	23.8207	-2.51795	0.297395	-8.4667	2.52E-17	1.67E-15
3221	*AHSG*	3.073391	-2.51597	0.545111	-4.61552	3.92E-06	2.22E-05
3388	*MCF2*	4.936475	-2.51151	0.555642	-4.52001	6.18E-06	3.34E-05
352	*IL12RB2*	40.55728	-2.5113	0.306808	-8.18524	2.72E-16	1.41E-14
10210	*TLX3*	1.042382	-2.50767	1.258305	-1.9929	0.046273	0.082833
1788	*CCL24*	6.86617	-2.5036	0.444625	-5.63081	1.79E-08	1.83E-07
6852	*PDX1*	39.65951	-2.50172	0.825238	-3.03151	0.002433	0.006491
59	*MT1X*	846.2607	-2.50114	0.232969	-10.7359	6.90E-27	2.14E-24
461	*CNTP3*	41.9304	-2.49942	0.319865	-7.81398	5.54E-15	2.19E-13
1796	*CC1*	41.97019	-2.49664	0.44421	-5.62041	1.91E-08	1.94E-07
12836	*ESX1*	0.567921	-2.49627	1.927073	-1.29537	0.195194	0.277934
2520	*B3GALT5*	6.009172	-2.49416	0.495683	-5.03176	4.86E-07	3.52E-06
410	*DHRS9*	218.5709	-2.49311	0.311956	-7.99187	1.33E-15	5.92E-14
6425	*PNPLA5*	2.401244	-2.48463	0.778117	-3.19314	0.001407	0.004003
11672	*ANXA8L1*	0.563655	-2.4842	1.540324	-1.61278	0.106793	0.167225
7003	*KLK8*	123.2171	-2.47867	0.832279	-2.97817	0.0029	0.007568
10953	*VGLL2*	0.98163	-2.47742	1.383305	-1.79094	0.073303	0.122318
918	*RHOV*	1614.712	-2.4729	0.36833	-6.71383	1.90E-11	3.77E-10
1397	*SFRP1*	223.6616	-2.4726	0.408001	-6.06027	1.36E-09	1.78E-08
5948	*GATA4*	7.382726	-2.46683	0.7286	-3.38572	0.00071	0.002181
132	*TMEM45A*	770.4886	-2.46416	0.258224	-9.54273	1.39E-21	1.93E-19
83	*IL1RN*	494.4384	-2.46221	0.243459	-10.1134	4.82E-24	1.05E-21
926	*NCR1*	3.324174	-2.46038	0.367223	-6.69995	2.08E-11	4.12E-10
332	*FPR2*	70.31933	-2.4597	0.29853	-8.23937	1.73E-16	9.53E-15
473	*CXCL11*	237.6209	-2.45958	0.31592	-7.78547	6.95E-15	2.68E-13
1786	*CNTP2*	152.0568	-2.457	0.435967	-5.63575	1.74E-08	1.78E-07
1128	*VTN*	47.29079	-2.45461	0.383651	-6.39802	1.57E-10	2.55E-09
6671	*SERPI4*	89.9527	-2.45404	0.791069	-3.10217	0.001921	0.005263
11425	*NBPF6*	0.617029	-2.44997	1.461809	-1.67599	0.093741	0.149961
989	*ENTHD1*	6.504275	-2.44711	0.37158	-6.58571	4.53E-11	8.37E-10
1584	*FGF12*	108.4482	-2.44208	0.418225	-5.83914	5.25E-09	6.05E-08
2051	*TRIM55*	16.95019	-2.4411	0.453821	-5.37899	7.49E-08	6.68E-07
1672	*MYEOV*	568.0101	-2.44082	0.424148	-5.75465	8.68E-09	9.49E-08
12100	*OR52E2*	0.54611	-2.43993	1.633834	-1.49338	0.135339	0.204429
1408	*KCNG1*	15.76224	-2.43571	0.402917	-6.04519	1.49E-09	1.94E-08
939	*F5*	381.4932	-2.4306	0.36378	-6.68152	2.36E-11	4.60E-10
178	*PCDH7*	714.0086	-2.42874	0.263277	-9.22504	2.83E-20	2.91E-18
955	*CLEC6A*	5.181365	-2.42653	0.364505	-6.65706	2.79E-11	5.35E-10
5225	*IGF2BP1*	336.2292	-2.42604	0.660363	-3.6738	0.000239	0.000836
180	*IL23A*	54.19261	-2.4257	0.263326	-9.21178	3.21E-20	3.26E-18
4871	*RFPL4B*	0.863174	-2.42087	0.631168	-3.83555	0.000125	0.00047
1840	*VIPR2*	43.24566	-2.41865	0.433633	-5.57765	2.44E-08	2.42E-07
3866	*LGALS9B*	5.998195	-2.41721	0.564449	-4.28243	1.85E-05	8.74E-05
9235	*SPANXB2*	0.860622	-2.41507	1.06277	-2.27243	0.023061	0.045639
372	*MGC87042*	79.61226	-2.41442	0.297227	-8.12315	4.54E-16	2.23E-14
452	*UBD*	1967.78	-2.4104	0.307639	-7.83516	4.68E-15	1.89E-13
381	*EPHB6*	147.5509	-2.40992	0.298239	-8.08047	6.45E-16	3.09E-14
7483	*TM4SF5*	11.36795	-2.40031	0.851137	-2.82013	0.0048	0.011726

2111	*CCL15*	20.62605	-2.40009	0.449743	-5.33657	9.47E-08	8.20E-07
3244	*CDHR5*	15.42281	-2.39506	0.520302	-4.60321	4.16E-06	2.34E-05
2619	*C21orf84*	3.342368	-2.39291	0.481488	-4.96981	6.70E-07	4.68E-06
4791	*NPSR1*	2.879156	-2.38818	0.616914	-3.87117	0.000108	0.000413
4242	*FAM55B*	1.880425	-2.38736	0.580961	-4.10933	3.97E-05	0.000171
5585	*CGB5*	9.121164	-2.38617	0.674646	-3.53692	0.000405	0.001325
10172	*NCR00200*	0.923427	-2.38469	1.189949	-2.00403	0.045067	0.080976
431	*IL8*	1446.861	-2.38076	0.300755	-7.91596	2.45E-15	1.04E-13
547	*FAM83A*	4462.663	-2.37986	0.316191	-7.52667	5.21E-14	1.74E-12
1242	*HAS1*	18.72372	-2.37735	0.380953	-6.24053	4.36E-10	6.42E-09
1240	*GAP43*	14.87996	-2.37584	0.380624	-6.24197	4.32E-10	6.37E-09
1588	*GJA3*	13.62416	-2.36613	0.405332	-5.8375	5.30E-09	6.10E-08
79	*SMOX*	474.8998	-2.36588	0.232094	-10.1937	2.12E-24	4.90E-22
1987	*C4orf26*	2.720132	-2.36553	0.435868	-5.42716	5.73E-08	5.27E-07
102	*DF5*	364.5393	-2.36549	0.237708	-9.95125	2.49E-23	4.46E-21
787	*HMSD*	6.646296	-2.362	0.338904	-6.96952	3.18E-12	7.37E-11
128	*HAS2*	140.2645	-2.3613	0.245525	-9.61734	6.76E-22	9.65E-20
6528	*KRT20*	2.34184	-2.35773	0.746277	-3.15932	0.001581	0.004427
275	*AQP9*	337.2869	-2.34946	0.277162	-8.47687	2.31E-17	1.54E-15
844	*HES2*	143.6084	-2.3485	0.342313	-6.86066	6.85E-12	1.48E-10
279	*CSTA*	220.5628	-2.346	0.277314	-8.45973	2.68E-17	1.76E-15
253	*TMEM71*	92.85614	-2.34097	0.27176	-8.61413	7.05E-18	5.09E-16
261	*GPC6*	612.9558	-2.33635	0.273631	-8.53832	1.36E-17	9.54E-16
314	*CXCL10*	917.4472	-2.33519	0.281113	-8.30692	9.82E-17	5.71E-15
563	*HAS2AS*	7.258763	-2.33433	0.312334	-7.47383	7.79E-14	2.53E-12
1728	*LOC400696*	13.50569	-2.33338	0.409441	-5.69894	1.21E-08	1.28E-07
64	*GFPT2*	451.3256	-2.33236	0.218876	-10.6561	1.63E-26	4.66E-24
2239	*LOC285696*	4.70641	-2.33206	0.44567	-5.2327	1.67E-07	1.36E-06
11410	*OR8S1*	0.568003	-2.33173	1.389022	-1.67869	0.093213	0.149321
13268	*CT45A6*	0.885873	-2.32912	1.964317	-1.18571	0.235735	0.324731
9124	*GABRG2*	7.832425	-2.32791	1.010518	-2.30368	0.02124	0.042548
41	*SRGN*	4265.535	-2.32488	0.202605	-11.4749	1.76E-30	7.86E-28
4147	*NKX2-3*	5.886608	-2.3241	0.55979	-4.15174	3.30E-05	0.000145
901	*HTR1D*	63.32163	-2.32408	0.344394	-6.74831	1.50E-11	3.03E-10
875	*TIMP4*	18.25896	-2.32122	0.341563	-6.79586	1.08E-11	2.25E-10
121	*VEGFC*	308.0714	-2.32088	0.239706	-9.68218	3.59E-22	5.42E-20
1742	*SERPI3*	3014.44	-2.31879	0.408255	-5.67974	1.35E-08	1.42E-07
802	*SLAMF9*	28.15767	-2.31751	0.333592	-6.94715	3.73E-12	8.49E-11
2663	*OSTBETA*	25.25095	-2.31684	0.468381	-4.94649	7.56E-07	5.18E-06
9242	*CTAG1B*	10.85328	-2.31624	1.020473	-2.26977	0.023221	0.045922
5152	*HBG1*	1.671003	-2.31466	0.62372	-3.71106	0.000206	0.000732
4030	*ASMT*	1.611355	-2.31044	0.549232	-4.20667	2.59E-05	0.000118
7844	*FGF23*	0.694959	-2.31026	0.853007	-2.70837	0.006761	0.015755
3250	*ART3*	1.653934	-2.30026	0.500323	-4.59755	4.27E-06	2.40E-05
492	*CCL21*	1456.352	-2.29839	0.297541	-7.72464	1.12E-14	4.17E-13
1950	*AQPEP*	6.868599	-2.29379	0.420455	-5.45549	4.88E-08	4.58E-07
1520	*MGC45800*	12.77054	-2.29235	0.388415	-5.90181	3.60E-09	4.32E-08
300	*CD274*	124.4587	-2.29088	0.273623	-8.37239	5.65E-17	3.44E-15
2551	*CARD17*	0.831019	-2.28715	0.456322	-5.01214	5.38E-07	3.86E-06
95	*SERPINE1*	2960.073	-2.28655	0.228277	-10.0166	1.29E-23	2.50E-21
1029	*ANK1*	56.12376	-2.28219	0.349265	-6.53426	6.39E-11	1.13E-09
8596	*TRDN*	3.309879	-2.28061	0.923877	-2.46852	0.013567	0.028847
2452	*ALDH1L1*	105.5522	-2.2772	0.448348	-5.0791	3.79E-07	2.83E-06
706	*C16orf74*	146.0827	-2.2741	0.31911	-7.12639	1.03E-12	2.67E-11
1285	*RP1L1*	7.832108	-2.27331	0.366997	-6.19435	5.85E-10	8.32E-09
1737	*NPY1R*	55.32077	-2.2712	0.399421	-5.68623	1.30E-08	1.37E-07
1273	*TMPRSS3*	371.7454	-2.26747	0.365677	-6.20074	5.62E-10	8.07E-09
1089	*MT1G*	133.0295	-2.26642	0.351545	-6.44703	1.14E-10	1.91E-09
2137	*KLRC4*	5.811016	-2.26438	0.425897	-5.31674	1.06E-07	9.04E-07
523	*MFI2*	410.6519	-2.26397	0.297085	-7.62062	2.52E-14	8.82E-13
5299	*SLCO1B3*	225.8363	-2.26316	0.619918	-3.65074	0.000261	0.000902
7625	*SERPINB11*	2.427331	-2.26175	0.814435	-2.77708	0.005485	0.013147
1602	*CYP4F12*	23.40723	-2.2591	0.388244	-5.81876	5.93E-09	6.76E-08

1302	*WDR69*	50.91771	-2.25818	0.365658	-6.17566	6.59E-10	9.25E-09
1501	*MMP1*	2839.682	-2.25774	0.381048	-5.92509	3.12E-09	3.80E-08
531	*KRT86*	102.4393	-2.25771	0.29766	-7.58485	3.33E-14	1.15E-12
1622	*CCL7*	29.63676	-2.25768	0.388991	-5.80392	6.48E-09	7.30E-08
723	*CCL26*	10.44696	-2.24752	0.316495	-7.10127	1.24E-12	3.12E-11
1092	*LOC100131726*	60.45346	-2.24474	0.348333	-6.44423	1.16E-10	1.95E-09
6717	*CDH7*	1.417051	-2.24455	0.728037	-3.08302	0.002049	0.005576
2596	*CLCA3P*	2.304142	-2.24414	0.450578	-4.98057	6.34E-07	4.46E-06
1251	*LAMA1*	111.7611	-2.24153	0.359612	-6.23317	4.57E-10	6.68E-09
646	*SDCBP2*	737.5875	-2.23745	0.308344	-7.25633	3.98E-13	1.13E-11
159	*LOXL2*	1795.279	-2.23526	0.238363	-9.37754	6.75E-21	7.80E-19
1138	*MMP12*	669.1322	-2.23034	0.349342	-6.38439	1.72E-10	2.76E-09
1576	*CD300E*	8.379792	-2.22497	0.380643	-5.84528	5.06E-09	5.86E-08
1688	*PITX1*	426.5339	-2.21939	0.386435	-5.74323	9.29E-09	1.01E-07
50	*CTSL1*	5605.085	-2.21681	0.20167	-10.9922	4.16E-28	1.52E-25
847	*MT1L*	82.67768	-2.21505	0.323284	-6.85171	7.30E-12	1.57E-10
6504	*FAM133A*	135.6455	-2.21429	0.699216	-3.16682	0.001541	0.004331
7543	*HHATL*	3.377552	-2.21377	0.789397	-2.80438	0.005041	0.012215
8939	*TMPRSS11F*	0.586554	-2.21283	0.936098	-2.36388	0.018084	0.036976
2261	*TP63*	479.0447	-2.20857	0.423313	-5.21734	1.82E-07	1.47E-06
3674	*SH3GL3*	5.223506	-2.20615	0.504014	-4.37716	1.20E-05	5.98E-05
7333	*RAX*	0.867695	-2.20553	0.768604	-2.86953	0.004111	0.010246
9995	*PYDC1*	0.612285	-2.20335	1.073188	-2.05308	0.040064	0.073262
2096	*CXCL6*	103.5051	-2.2033	0.412035	-5.34736	8.92E-08	7.78E-07
4946	*LHX5*	2.138464	-2.20107	0.578955	-3.8018	0.000144	0.000531
3669	*PRKCG*	8.321616	-2.2004	0.502498	-4.37892	1.19E-05	5.94E-05
3882	*PITX3*	2.50428	-2.19704	0.513538	-4.27824	1.88E-05	8.87E-05
736	*CXCR1*	22.98721	-2.1964	0.310305	-7.07821	1.46E-12	3.63E-11
240	*SLC2A14*	44.99796	-2.19636	0.252066	-8.71343	2.95E-18	2.24E-16
9116	*SLC6A19*	0.645692	-2.19537	0.952091	-2.30585	0.021119	0.042343
6284	*KRT85*	0.713211	-2.19373	0.675093	-3.24953	0.001156	0.003362
1615	*GUCA1A*	6.37204	-2.19107	0.377186	-5.80898	6.29E-09	7.11E-08
9997	*TEX101*	0.94377	-2.18279	1.06334	-2.05277	0.040095	0.073303
2685	*WNT16*	11.12222	-2.18035	0.442352	-4.929	8.27E-07	5.63E-06
4709	*FETUB*	2.400952	-2.17919	0.55803	-3.90516	9.42E-05	0.000365
8792	*UGT2A3*	0.898042	-2.17675	0.903006	-2.41056	0.015928	0.033111
724	*CLEC4D*	8.813067	-2.17497	0.306334	-7.09999	1.25E-12	3.15E-11
1005	*KLHL4*	47.64362	-2.17046	0.330413	-6.56893	5.07E-11	9.22E-10
5213	*C9*	1.06361	-2.16923	0.58926	-3.68127	0.000232	0.000814
157	*PLOD2*	3069.44	-2.16761	0.230795	-9.3919	5.89E-21	6.86E-19
941	*HCG4*	54.41907	-2.16708	0.324419	-6.67986	2.39E-11	4.65E-10
8095	*AKR1C4*	10.91707	-2.16476	0.823503	-2.62872	0.008571	0.019351
3616	*LY6G6C*	3.71441	-2.16438	0.490965	-4.40842	1.04E-05	5.26E-05
14274	*FTHL17*	0.478183	-2.16285	2.324533	-0.93044	0.352141	0.450864
2541	*ZPLD1*	10.62158	-2.16077	0.430234	-5.02231	5.11E-07	3.67E-06
254	*CD163L1*	147.1852	-2.16041	0.251272	-8.59791	8.12E-18	5.84E-16
9812	*RIPPLY2*	0.849648	-2.15938	1.025529	-2.10562	0.035237	0.065637
681	*XCL2*	25.72791	-2.1564	0.300303	-7.18074	6.93E-13	1.86E-11
2453	*COL17A1*	1394.238	-2.15434	0.424194	-5.07868	3.80E-07	2.83E-06
6445	*FAM83C*	2.166798	-2.15413	0.675668	-3.18815	0.001432	0.00406
105	*ITGA6*	2691.616	-2.1522	0.217241	-9.90696	3.88E-23	6.76E-21
7081	*KC6*	1.587354	-2.1519	0.729472	-2.94994	0.003178	0.008204
345	*IL2RA*	173.2097	-2.15092	0.262037	-8.20849	2.24E-16	1.19E-14
756	*C15orf48*	1768.129	-2.1476	0.304811	-7.04569	1.85E-12	4.46E-11
249	*BEND6*	57.66929	-2.14482	0.247751	-8.65717	4.84E-18	3.55E-16
15147	*LGALS13*	0.47216	-2.14238	2.919554	-0.73381	0.463067	0.558756
12260	*ADIPOQ*	0.594102	-2.14227	1.48065	-1.44684	0.147941	0.220548
80	*B4GALT6*	119.5395	-2.14127	0.210416	-10.1764	2.53E-24	5.78E-22
970	*FBXL13*	26.24577	-2.13677	0.32219	-6.632	3.31E-11	6.24E-10
9538	*SLC2A2*	0.718959	-2.13398	0.977131	-2.18392	0.028968	0.055509
5773	*SPIC*	0.796574	-2.13298	0.617039	-3.4568	0.000547	0.001731
2459	*CYP11A1*	54.64595	-2.13283	0.420393	-5.07342	3.91E-07	2.90E-06
6038	*OR7A5*	1.000082	-2.13144	0.636608	-3.34812	0.000814	0.002463

8166	*NEUROG2*	0.686489	-2.12899	0.816696	-2.60684	0.009138	0.020453
4421	*SYT5*	27.84404	-2.12563	0.527321	-4.031	5.55E-05	0.00023
1484	*ADAM23*	22.80853	-2.12237	0.357246	-5.9409	2.83E-09	3.49E-08
1640	*C9orf169*	46.41813	-2.122	0.366796	-5.78523	7.24E-09	8.07E-08
1019	*CCL8*	117.3048	-2.11972	0.323863	-6.5451	5.95E-11	1.07E-09
12931	*ONECUT3*	0.520467	-2.11898	1.666697	-1.27136	0.2036	0.287773
2632	*CSF2*	51.03215	-2.1151	0.425918	-4.96599	6.84E-07	4.75E-06
11475	*MUC17*	0.677285	-2.11143	1.269213	-1.66357	0.096198	0.153221
7888	*LST-3TM12*	6.188785	-2.11055	0.783547	-2.69358	0.007069	0.016379
11606	*CARD18*	0.577648	-2.10575	1.290164	-1.63216	0.102646	0.161646
3451	*TGM5*	4.848572	-2.10564	0.469239	-4.48736	7.21E-06	3.82E-05
1903	*APCDD1L*	41.07815	-2.10109	0.381486	-5.50764	3.64E-08	3.49E-07
1213	*UNC5A*	28.65988	-2.09938	0.333937	-6.28674	3.24E-10	4.88E-09
2731	*SRD5A2*	45.37308	-2.09894	0.428883	-4.89396	9.88E-07	6.61E-06
8072	*RPL10L*	0.975001	-2.09662	0.795681	-2.635	0.008414	0.019051
1393	*TMEM171*	42.94473	-2.09419	0.345491	-6.06151	1.35E-09	1.77E-08
643	*CCL18*	2197.229	-2.09267	0.288211	-7.26089	3.85E-13	1.09E-11
3802	*LMO1*	3.444831	-2.09253	0.485188	-4.31282	1.61E-05	7.75E-05
91	*BCL2A1*	244.9128	-2.09244	0.208581	-10.0318	1.11E-23	2.20E-21
2125	*FAM196A*	38.76687	-2.09058	0.39245	-5.327	9.98E-08	8.59E-07
636	*EGLN3*	1444.523	-2.08897	0.287173	-7.27426	3.48E-13	1.00E-11
855	*GREM1*	1306.989	-2.08785	0.305308	-6.83851	8.00E-12	1.71E-10
593	*FCGR3B*	71.35821	-2.08686	0.282439	-7.38871	1.48E-13	4.57E-12
1163	*AREG*	908.4808	-2.08602	0.328837	-6.34364	2.24E-10	3.53E-09
1221	*PRR15*	185.6295	-2.08487	0.33235	-6.27312	3.54E-10	5.30E-09
7401	*ZNF280A*	4.813567	-2.0845	0.732361	-2.84628	0.004423	0.010924
895	*G0S2*	762.7118	-2.08432	0.308318	-6.76029	1.38E-11	2.81E-10
3398	*HPD*	12.19238	-2.08337	0.461406	-4.51527	6.32E-06	3.40E-05
2017	*VWDE*	76.77976	-2.08133	0.384993	-5.40614	6.44E-08	5.84E-07
326	*SEMA7A*	533.014	-2.07701	0.251616	-8.25471	1.52E-16	8.54E-15
1702	*SCN5A*	11.88273	-2.07492	0.362292	-5.72719	1.02E-08	1.10E-07
6143	*NPY5R*	2.632684	-2.07341	0.627504	-3.30422	0.000952	0.002834
668	*GZMB*	302.5927	-2.07187	0.287682	-7.20196	5.94E-13	1.62E-11
1062	*RIMKLB*	1323.003	-2.07141	0.319417	-6.48497	8.87E-11	1.53E-09
2754	*SLC28A3*	44.44211	-2.07131	0.424432	-4.88019	1.06E-06	7.03E-06
7379	*CALML3*	31.84949	-2.06951	0.724662	-2.85583	0.004292	0.010632
4328	*LGALS7*	18.78504	-2.06928	0.50859	-4.06866	4.73E-05	0.0002
530	*KLRD1*	67.3009	-2.06848	0.272586	-7.58837	3.24E-14	1.12E-12
74	*UBASH3B*	332.1942	-2.06815	0.200556	-10.3121	6.21E-25	1.53E-22
9017	*BCAR4*	1.586552	-2.06655	0.88402	-2.33768	0.019404	0.039331
18756	*GAGE13*	0.448544	-2.06556	2.320657	-0.89007	0.373426	NA
2762	*PCSK1*	64.96838	-2.06239	0.423017	-4.87543	1.09E-06	7.18E-06
976	*ANGPTL4*	1556.47	-2.05992	0.311263	-6.61794	3.64E-11	6.82E-10
7826	*RAD21L1*	0.953573	-2.05834	0.757756	-2.71636	0.0066	0.015415
2650	*DEFB1*	140.1731	-2.05063	0.413841	-4.95511	7.23E-07	4.99E-06
621	*IL1B*	187.295	-2.05017	0.280257	-7.3153	2.57E-13	7.56E-12
403	*FAM26F*	218.1342	-2.04729	0.255228	-8.02141	1.05E-15	4.74E-14
88	*GBP1*	2019.834	-2.04258	0.202842	-10.0698	7.51E-24	1.56E-21
837	*TMIGD2*	12.22645	-2.03098	0.295311	-6.87742	6.09E-12	1.33E-10
231	*PDCD1LG2*	134.5196	-2.03081	0.231896	-8.75741	2.00E-18	1.58E-16
915	*PHOSPHO1*	12.85874	-2.02846	0.301987	-6.71704	1.85E-11	3.70E-10
3232	*MMP10*	159.8261	-2.02753	0.439646	-4.61173	3.99E-06	2.26E-05
1392	*FAM196B*	5.650989	-2.02717	0.334422	-6.0617	1.35E-09	1.77E-08
8668	*SPDYC*	1.889456	-2.02495	0.827902	-2.44588	0.01445	0.030469
4112	*DIRC1*	1.024603	-2.0248	0.485715	-4.16871	3.06E-05	0.000136
263	*LILRA5*	75.29485	-2.01835	0.236434	-8.53663	1.38E-17	9.60E-16
13263	*GAGE2A*	5.871625	-2.01637	1.699614	-1.18637	0.235475	0.324495
18278	*A11*	0.43284	-2.01633	2.608443	-0.773	0.439521	NA
67	*APOL1*	4757.397	-2.01213	0.191294	-10.5185	7.10E-26	1.94E-23
84	*KLF4*	763.36	-2.00688	0.19844	-10.1133	4.83E-24	1.05E-21
458	*GZMA*	281.523	-2.00623	0.256565	-7.81956	5.30E-15	2.12E-13
239	*OASL*	319.7161	-2.00599	0.230223	-8.71327	2.95E-18	2.24E-16
4881	*CRLF2*	6.060376	-2.00514	0.523285	-3.83183	0.000127	0.000476

6141	*GTSF1*	90.09574	-2.00479	0.606679	-3.30454	0.000951	0.002831
5665	*SNX31*	2.083521	-2.0029	0.571777	-3.50294	0.00046	0.001485
1739	*IFNG*	22.02397	-2.00244	0.352352	-5.68305	1.32E-08	1.39E-07
5403	*KRT12*	1.142915	-2.00123	0.554888	-3.60654	0.00031	0.001049
5400	*LOC642587*	56.94264	-2.00087	0.554662	-3.60736	0.000309	0.001047

Table S6. A list of cytokines in pathway: 'KEGG cytokine-cytokine receptor interaction' (n = 265 genes) analyzed in this study.

ACVR1	*CCR5*	*EDAR*	*IL10RB*	*IL4R*	*TGFB3*
ACVR1B	*CCR6*	*EGF*	*IL11*	*IL5*	*TGFBR1*
ACVR2A	*CCR7*	*EGFR*	*IL11RA*	*IL5RA*	*TGFBR2*
ACVR2B	*CCR8*	*EPO*	*IL12A*	*IL6*	*TNF*
ACVRL1	*CCR9*	*EPOR*	*IL12B*	*IL6R*	*TNFRSF10A*
AMH	*CD27*	*FAS*	*IL12RB1*	*IL6ST*	*TNFRSF10B*
AMHR2	*CD40*	*FASLG*	*IL12RB2*	*IL7*	*TNFRSF10C*
BMP2	*CD40LG*	*FLT1*	*IL13*	*IL7R*	*TNFRSF10D*
BMP7	*CD70*	*FLT3*	*IL13RA1*	*IL9*	*TNFRSF11A*
BMPR1A	*CLCF1*	*FLT3LG*	*IL15*	*IL9R*	*TNFRSF11B*
BMPR1B	*CNTF*	*FLT4*	*IL15RA*	*INHBA*	*TNFRSF12A*
BMPR2	*CNTFR*	*GDF5*	*IL17A*	*INHBB*	*TNFRSF13B*
CCL1	*CRLF2*	*GH1*	*IL17B*	*INHBC*	*TNFRSF13C*
CCL11	*CSF1*	*GH2*	*IL17RA*	*INHBE*	*TNFRSF14*
CCL13	*CSF1R*	*GHR*	*IL17RB*	*KDR*	*TNFRSF17*
CCL14	*CSF2*	*HGF*	*IL18*	*KIT*	*TNFRSF18*
CCL15	*CSF2RA*	*IFNA1*	*IL18R1*	*KITLG*	*TNFRSF19*
CCL16	*CSF2RB*	*IFNA10*	*IL18RAP*	*LEP*	*TNFRSF1A*
CCL17	*CSF3*	*IFNA13*	*IL19*	*LEPR*	*TNFRSF1B*
CCL18	*CSF3R*	*IFNA14*	*IL1A*	*LIF*	*TNFRSF21*
CCL19	*CTF1*	*IFNA16*	*IL1B*	*LIFR*	*TNFRSF25*
CCL2	*CX3CL1*	*IFNA17*	*IL1R1*	*LTA*	*TNFRSF4*
CCL20	*CX3CR1*	*IFNA2*	*IL1R2*	*LTB*	*TNFRSF6B*
CCL21	*CXCL1*	*IFNA21*	*IL1RAP*	*LTBR*	*TNFRSF8*
CCL22	*CXCL10*	*IFNA4*	*IL2*	*MET*	*TNFRSF9*
CCL23	*CXCL11*	*IFNA5*	*IL20*	*MPL*	*TNFSF10*
CCL24	*CXCL12*	*IFNA6*	*IL20RA*	*NGFR*	*TNFSF11*
CCL25	*CXCL13*	*IFNA7*	*IL20RB*	*OSM*	*TNFSF12*
CCL26	*CXCL14*	*IFNA8*	*IL21*	*OSMR*	*TNFSF13*
CCL27	*CXCL16*	*IFNAR1*	*IL21R*	*PDGFA*	*TNFSF13B*
CCL28	*CXCL2*	*IFNAR2*	*IL22*	*PDGFB*	*TNFSF14*
CCL3	*CXCL3*	*IFNB1*	*IL22RA1*	*PDGFC*	*TNFSF15*
CCL3L1	*CXCL5*	*IFNE*	*IL22RA2*	*PDGFRA*	*TNFSF18*
CCL3L3	*CXCL6*	*IFNG*	*IL23A*	*PDGFRB*	*TNFSF4*
CCL4	*CXCL8*	*IFNGR1*	*IL23R*	*PF4*	*TNFSF8*

CCL4L2	*CXCL9*	*IFNGR2*	*IL24*	*PF4V1*	*TNFSF9*
CCL5	*CXCR1*	*IFNK*	*IL25*	*PLEKHO2*	*TPO*
CCL7	*CXCR2*	*IFNL1*	*IL26*	*PPBP*	*TSLP*
CCL8	*CXCR3*	*IFNL2*	*IL2RA*	*PPBPP1*	*VEGFA*
CCR1	*CXCR4*	*IFNL3*	*IL2RB*	*PRL*	*VEGFB*
CCR10	*CXCR5*	*IFNLR1*	*IL2RG*	*PRLR*	*VEGFC*
CCR2	*CXCR6*	*IFNW1*	*IL3*	*RELT*	*VEGFD*
CCR3	*EDA*	*IL10*	*IL3RA*	*TGFB1*	*XCL1*
CCR4	*EDA2R*	*IL10RA*	*IL4*	*TGFB2*	*XCL2*
